# Investigating Ultrafiltration Membranes and Operation Modes for Improved Lentiviral Vector Processing

**DOI:** 10.1002/elsc.202400057

**Published:** 2025-01-03

**Authors:** Jennifer J. Labisch, Maria Evangelopoulou, Tobias Schleuß, Andreas Pickl

**Affiliations:** ^1^ Lab Essentials Applications Development Sartorius Göttingen Germany; ^2^ Institute of Technical Chemistry Leibniz University Hannover Hannover Germany; ^3^ Ultrafiltration Membrane Technology Sartorius Göttingen Germany

**Keywords:** centrifugal ultrafilters, crossflow cassettes, downstream processing, lentiviral vectors, stirred cells, tangential flow filtration, ultrafiltration

## Abstract

The demand for lentiviral vectors (LVs) as tools for ex vivo gene therapies is ever‐increasing. Despite their promising applications, challenges in LV production remain largely due to the fragile envelope, which challenges the maintenance of vector stability. Thus, downstream processing optimization to enhance efficiency, yield, and product quality is necessary. This study investigated the influence of membrane types and filtration devices during ultrafiltration (UF). Nine different membrane materials consisting of polyethersulfone (PES), regenerated cellulose, or Hydrosart, with distinct molecular weight cutoffs, were evaluated in stirred cells, centrifugal ultrafilters, and crossflow cassettes. The evaluation was based on the ability to retain infectious LV particles and remove impurities. The analysis revealed that a reinforced 100 kDa PES and a 300 kDa Hydrosart membrane had the best overall ability to concentrate infectious LVs and remove DNA, especially when operated in a stirred cell. Challenges were seen in the nonoptimized crossflow cassette process, where infectious LV recovery was generally lower compared to other devices. We demonstrated that membrane material and filtration device have a direct impact on the efficiency of LV UF.

## 1 Introduction

Third‐generation, self‐inactivating lentiviral vectors (LVs) have been optimized over the past two decades for clinical use in gene transfer to target cells, like T cells or hematopoietic stem cells. A major advantage is their ability to infect nondividing and dividing cells and achieve stable, long‐term integration of the transgene into the host genome, ensuring its sustained expression [1]. To date, eight drug products using LVs have been approved, including five gene‐modified cell therapy and three gene therapy products (https://srtrs.info/FDA_Approved_Cellular_and_Gene_Therapy_Products). With many more LVs in the pipeline currently undergoing clinical phases [2], the need for effective and cost‐efficient LV manufacturing is increasing. Despite their promising applications, challenges in LV manufacturing persist primarily due to the fragile envelope, which complicates the maintenance of vector functionality. An improvement in the manufacturing process of LVs is thus essential to meet the demand [3].

**Summary**

• The research on lentiviral vector (LV) bioprocessing highlights the critical need for optimizing ultrafiltration (UF) processes to enhance the efficiency of LV production.

• By systematically screening polyethersulfone and cellulose‐based membranes with different molecular weight cutoffs (MWCOs) in various UF devices, this study provides valuable insights into selecting the most suitable membrane materials and UF strategies for handling fragile, enveloped viral vectors.

• The findings emphasize that the performance of UF membranes varies with material, MWCO, and device, necessitating tailored optimization for each process. This research is instrumental for both laboratory‐scale and industrial‐scale LV production, aiding in the development of more effective and reliable UF protocols.

• Moreover, it underscores the importance of thorough membrane screening due to variability in MWCO consistency across different manufacturers, ultimately contributing to more precise and efficient bioprocessing of LVs and other large, enveloped vectors.

Downstream processing (DSP) of LVs involves various unit operations tailored to specific applications. For instance, in early research simple clarification and concentration steps may suffice to achieve adequate LV titer and purity for animal studies [4, 5, 6]. However, clinical applications demand higher purity, adding more complexity to the DSP. Ultrafiltration (UF) is a commonly used, scalable, robust, and cost‐effective method for concentration or buffer exchange (diafiltration) [7, 8], utilized after the clarification process during which cells and cell debris are removed, or after the purification step, typically employing chromatography [9, 10]. Besides UF, clarification with microfilters or depth filters [11], chromatography using membrane adsorbers, and sterilizing filtration are membrane processes employed for LV DSP [11]. During UF, macromolecules in solution are separated by size using a semipermeable membrane with a defined molecular weight cutoff (MWCO), indicating the retention of 90% of molecules at or above this molecular weight [12, 13]. For viruses, MWCO selection depends on viral diameter rather than molecular weight. For UF of LVs, membranes with an MWCO of 100–750 kDa have been employed [8]. The asymmetric UF membranes comprise a thin selective skin layer with small pores at the top and a highly porous, thick support layer for mechanical stability [14]. Common polymer materials for UF membranes include polyethersulfone (PES), polyvinylidene fluoride (PVDF), and regenerated cellulose (RC), among others [13]. Membranes can be reinforced with a fleece to improve tensile strength and resistance to tearing or puncturing [15]. PES membranes offer thermal and pH stability but can be prone to unspecific binding and fouling due to their hydrophobicity [16, 17]. Although chemically resistant and usable across a broad pH spectrum, PVDF membranes are expected to face restrictions in the EU due to its environmental persistence and potential health risks as a polyfluorinated alkyl substances (PFASs) [18]. Cellulose‐based membranes are hydrophilic and less prone to fouling, have high tensile strength, but have limited temperature and pH stability [19]. However, crosslinking of the cellulose membrane (Hydrosart membrane) reduces swelling and enables operation in basic pH conditions [7].

Besides membrane material selection, filtration mode, process parameters (e.g., flux, transmembrane pressure [TMP]), and device design (e.g., membrane area) also impact product quality and yield [20]. UF can be performed in dead‐end (normal flow) and tangential flow filtration (TFF) modes. In dead‐end filtration, the feed flows perpendicularly through the membrane potentially leading to filter‐cake formation which reduces flow rate over time. In contrast, crossflow or TFF allows the feed to flow parallel to the membrane, splitting into retentate and permeate streams, and the retentate is circulated back to the membrane. Although TFF also experiences membrane fouling, it maintains a more consistent flow rate over time [21].

Various UF devices are available depending on the process volume. Lab‐scale devices comprise centrifugal ultrafilters (up to 20 mL) and stirred cells (10–400 mL), both operated in dead‐end mode. For larger volumes, TFF setups, such as flat sheet cassettes or hollow fiber membranes, are preferred [22]. Compared with other lab‐scale concentration methods like ultracentrifugation (often used in early LV research), or chemical precipitation, UF offers advantages: it requires no sample pretreatment, is less time‐consuming, and can be scaled to process scale using TFF mode [23]. Although protein bioprocessing is well‐studied, viral vector bioprocessing remains relatively unexplored especially given the varied physicochemical properties of different vectors. A previous study on protein UF has shown different retention behaviors of various proteins when using stirred cells or crossflow systems with the same membrane [24]. However, the influences of different membrane materials and process conditions have not yet been sufficiently analyzed for viral vectors.

In this study, we report the screening of different membrane materials and MWCOs for the UF of clarified LVs in different UF devices and therefore filtration modes. We examined nine different membranes made of PES, RC, or Hydrosart, all with MWCOs of 100 and 300 kDa and one additional PES membrane with 1000 kDa. These membranes were incorporated in centrifugal ultrafilters, stirred cells, and crossflow cassettes. We thereby determined the impact of the UF membranes on the process performance and product quality depending on the filtration mode.

## 2 Materials and Methods

### 2.1 LV Production, Harvest, and Clarification

Third‐generation LVs, which carry a GFP reporter gene, were produced by transient transfection of suspension HEK293T/17 SF cells (ACS‐4500, ATCC) with four plasmids (Aldevron, USA) in a UniVessel 10 L bioreactor (Sartorius, Germany) operated by a BIOSTAT B‐DCU (Sartorius). LV production, harvest, and nucleic acid digestion with all materials used are described in detail in Labisch et al. [25]. The LV containing cell culture broth was directly clarified using Sartoclear Dynamics Lab V50 (0.45 µm PES membrane version) with 5 g L^−1^ diatomaceous earth (Sartorius, Germany) and a Microsart e.jet vacuum pump (Sartorius, Germany). The LV was aliquoted and stored at −80°C until use for UF experiments. If not indicated otherwise, the same LV batch was used for all UF experiments for better comparability. The clarified LV batch (starting material) had a mean infectious LV recovery of 1 × 10^8^ TU mL^−1^ as well as an averaged DNA and protein concentration of 502 ng mL^−1^ and 196 µg mL^−1^, respectively.

### 2.2 Ultrafiltration

An extensive investigation of how membrane material influences LV UF was performed using nine different UF membranes from Sartorius. These membranes varied in material composition, either PES or cellulose‐based (RC or Hydrosart), and had different MWCOs (100, 300, and 1000 kDa). Moreover, the 100 and 300 kDa PES membranes were tested both with and without fleece reinforcement. The same membrane batch for each membrane type was used for all experiments. Refer to Table 1 for a comprehensive list of membranes and their specifications.

**TABLE 1:** Overview of membranes used for UF.

Membrane material	MWCO (kDa)	Thickness (µm)	Specifications
PES	100	262.95 ± 41.72	Fleece reinforced (+R)
PES	100	127.22 ± 6.21	Not reinforced
PES	300	262.18 ± 33.34	Fleece reinforced (+R)
PES	300	128.07 ± 7.91	Not reinforced
PES	1000	250	Fleece reinforced
RC	100	247.29 ± 9.78	Fleece reinforced
RC	300	241.67 ± 8.4	Fleece reinforced
Hydrosart	100	217.82 ± 9.04	Fleece reinforced
Hydrosart	300	213.22 ± 10.42	Fleece reinforced

*Note:* Thickness given in mean ± standard deviation.

Abbreviations: MWCO, molecular weight cutoff; PES, polyethersulfone; RC, regenerated cellulose; UF, ultrafiltration.

Three different devices were used for UF experiments to evaluate membrane performance: stirred cells, centrifugal ultrafilters, and the multiparallel small‐scale crossflow filtration system Ambr Crossflow system.

Each device operates differently, either in a dead‐end or TFF mode, adding complexity to the filtration process. Thus, it was aimed to compare the different UF membranes in the distinct devices as they may behave differently depending on the mode of operation. Four replicate experiments for each membrane in every device were performed. More details on experimental specifications are depicted in Table 2. The stirred cell had a lower loading density due to its 10 mL capacity. We did not reduce the volume in the centrifugal concentrators to match this density, as users typically prefer to maximize capacity for higher concentration. To better reflect real use, we loaded the concentrators with 15 mL. The crossflow cassette was loaded with the same density as the concentrator to challenge the membrane.

**TABLE 2:** Experimental specifications for the three devices used for UF.

Device	Effective filtration area (cm^2^)	Loading LV volume (mL)	Loading density (L m^−^^2^)	Retentate volume (mL)	Volumetric concentration factor
Centrifugal concentrator	3.90	15	38.46	2.0	7.50
Stirred cell	3.80	10	26.32	1.5	6.67
Crossflow cassettes	10.52	40	38.04	6.0	6.67

Abbreviations: LV, lentiviral vector; UF, ultrafiltration.

The clarified LV suspension (starting material) was thawed and kept on ice until the start of the experiment. An individual starting material sample was collected for each parallel running filtration and kept at room temperature during the filtration. The retentate volumes were determined by weighing. Collected samples were immediately transferred on ice and stored at −80°C until analytical evaluation.

#### 2.2.1 Centrifugal Concentrators

Before evaluating the nine different membranes in the centrifugal ultrafilters, PES and RC membranes with 100, 300, or 1000 kDa MWCO from various vendors were tested to determine optimal process conditions and compare commercially available products. The centrifuge Multifuge X1R (Thermo Fisher Scientific, USA) with a swing‐out rotor was used. All centrifugal ultrafilters were washed once with phosphate‐buffered saline (PBS) before loading 15 mL of clarified LV solution. Centrifugation was stopped several times to monitor volume reduction and to rinse the membrane using a pipette. Optimal process conditions were identified with selected centrifugal ultrafilters, therefore, 500, 1000, and 2000 × *g* were applied, and the volumetric concentration factor was varied (exact concentration factors listed in Section 3). Afterward, eight centrifugal ultrafilters from different manufacturers (Table 3) were tested in triplicates at 500 × *g* with a volumetric concentration factor of 7.5.

**TABLE 3:** Centrifugal concentrators with membrane material and molecular weight cutoff (MWCO).

Product	Manufacturer	Membrane material	MWCO (kDa)	Effective filtration area (cm^2^)
Vivaspin Turbo 15	Sartorius, Germany	RC	100	8.6
Vivaspin Turbo 15	Sartorius, Germany	PES	100	8.6
Vivaspin 20	Sartorius, Germany	PES	100	6.0
Vivaspin 20	Sartorius, Germany	PES	300	6.0
Vivaspin 20	Sartorius, Germany	PES	1000	6.0
Amicon Ultra‐15	Merck Millipore, USA	RC	100	7.6
Macrosep Advance	Cytiva (formerly Pall), USA	PES	100	7.2

Abbreviations: PES, polyethersulfone; RC, regenerated cellulose.

The shape and design of centrifugal ultrafilters can also influence the process performance. To better compare different UF membranes independent of the design of the centrifugal ultrafilter, Vivaspin 15R was custom‐made by incorporating nine different membranes (Table 1) into the same device. UF was operated at 500 × *g*, further specifications are listed in Table 2.

#### 2.2.2 Stirred Cell

The stirred cells feature a cell body that can load up to 10 mL of sample. A magnetic stirrer can be placed inside to float above the membrane. Membranes with a diameter of 25 mm were secured with an O‐ring on the membrane holder. A cap with an integral pressure inlet sealed the unit, enabling air introduction for pressure‐driven UF. A pressure of 2 bar was applied. First, the effect of stirring speed was investigated using a different LV batch produced under the same process conditions outlined in Section 2.1. Stirring speeds of 0, 400, and 800 rpm were tested with the 300 kDa PES fleece‐reinforced membrane. Further experiments using the whole range of UF membranes (Table 1) were performed at 800 rpm. Further specifications are listed in Table 2. Membranes were flushed with 10 mL PBS before loading with LV solution. Between runs stirred cells were cleansed with 0.1 M NaOH and dH_2_O.

#### 2.2.3 Crossflow Cassettes

To enable TFF, the Ambr Crossflow system (Sartorius, Germany) was used. This system comprises four crossflow channels, which can be operated in parallel. Custom‐built filter cassettes were used to accommodate a mesh support layer on the permeate side and one layer of each membrane (Table 1), which were cut to size using a roller press and a respective punching template. Before filtration, the feed lines were flushed with 1 M NaOH and dH_2_O to minimize contamination. The cassettes were equilibrated with 40 mL of PBS, and a gas tightness test was performed. UF was performed with a TMP of 500 mbar and a flow rate of 10 mL min^−1^. The process parameters were chosen based on a literature review [26, 27, 28, 29]. Further specifications are listed in Table 2. The system was cleaned with 1 M NaOH followed by dH_2_O.

### 2.3 Analytics

#### 2.3.1 Determination of Infectious LV Titer

The infectious LV titer was quantified via real‐time imaging using the Incucyte S3 live‐cell analysis system (Sartorius, Germany). Adherent HEK293T cells (ACC 635, DSMZ) were infected with serially diluted LV samples, and GFP expression was measured as described in detail in Labisch et al. [30] with the following modifications: no staining was performed, and transgene expression (GFP) was read out 60 h postinfection. Samples were analyzed in duplicates.

#### 2.3.2 Determination of LV Particle Titer

The LV particle titer was quantified by performing a p24 enzyme‐linked immunosorbent assay (ELISA) using the QuickTiter Lentivirus titer kit (Cell Biolabs, USA). The absorbance at 450 nm of the samples correlated with the concentration of the p24 capsid protein. The standard curve obtained was fitted by a second‐degree polynomial. The p24 concentrations determined were converted into viral particle titers by assuming that 1.25 × 10^7^ LV particles contain 1 ng of p24 and 1 LV particle contains about 2000 molecules of p24 [31].

#### 2.3.3 Total dsDNA Quantification

The concentration of total dsDNA was determined using the Quant‐iT Pico‐Green dsDNA assay (Thermo Fisher Scientific, USA) according to the manufacturer's instructions. Standards and samples were assessed in duplicates in black 96‐well microtiter plates (Corning, USA). The samples were excited at 480 nm, and the fluorescence emission intensity was measured at 520 nm using a FLUOstar Omega plate reader (BMG Labtech, Germany). The standard curve was fitted by linear regression.

#### 2.3.4 Total Protein Measurement

According to the manufacturer's instructions, the total protein concentration was determined with the Pierce Coomassie Bradford protein assay kit (Thermo Fisher Scientific, USA). Standards and samples were analyzed in duplicates in transparent 96‐well microtiter plates (Greiner Bio‐one, Austria). The absorbance was read at 595 nm with a FLUOstar Omega plate reader (BMG Labtech, Germany). A second‐degree polynomial was used to fit the standard curve.

#### 2.3.5 Statistical Analysis

Statistical analysis and graphing of the data were performed using GraphPad Prism. For experiments with *N* ≥ 3 replicates, means and standard deviations were calculated. For the comparison of membranes within one device, a one‐way analysis of variance (ANOVA) was performed, and for the comparison of a membrane across all devices, a two‐way ANOVA was performed, followed by a Tukey test. Statistical significance was determined at *p* ≤ 0.05, and significance levels were declared as follows *p* value ≤ 0.05 (*), *p* values ≤ 0.01 (**), and *p* value < 0.001 (***). Statistical significance in figures is shown exemplarily for better clarity and where relations between results and membrane material are discussed. Full statistical results are available in the supplementary section (Tables S1–S16).

## 3 Results

### 3.1 Centrifugal Ultrafiltration—A Straightforward Standard Laboratory Method or Is There More to Consider?

Centrifugal ultrafilters are well‐known laboratory consumables used to concentrate and rebuffer organic and inorganic macromolecules and designed to fit into standard centrifuge tubes. They are so commonplace in everyday laboratory work that it is easy to avoid giving much thought to process optimization when selecting and using them. A centrifugal ultrafilter is chosen based on the sample volume (µL to mL range) and the sample properties, mainly the molecular weight or size of the target molecule. The sample is loaded into the upper chamber of the centrifugal ultrafilter and the centrifugal force drives the liquid through a semipermeable membrane retaining and concentrating the target molecule, as the volume reduces. The process is stopped when the desired retentate volume is reached.

PES and RC membranes with 100, 300, or 1000 kDa MWCO from various vendors were tested to determine the optimal centrifugal force and concentration factor. Seven ultrafilters from different manufacturers were then tested under the determined optimal conditions. Finally, Vivaspin 15R devices were custom‐made with nine different membranes (Table 1) to compare UF membranes independently of concentrator design.

#### 3.1.1 Optimization of Process Conditions for Lentiviral Concentration Using Centrifugal Ultrafiltration

**FIGURE 1:**
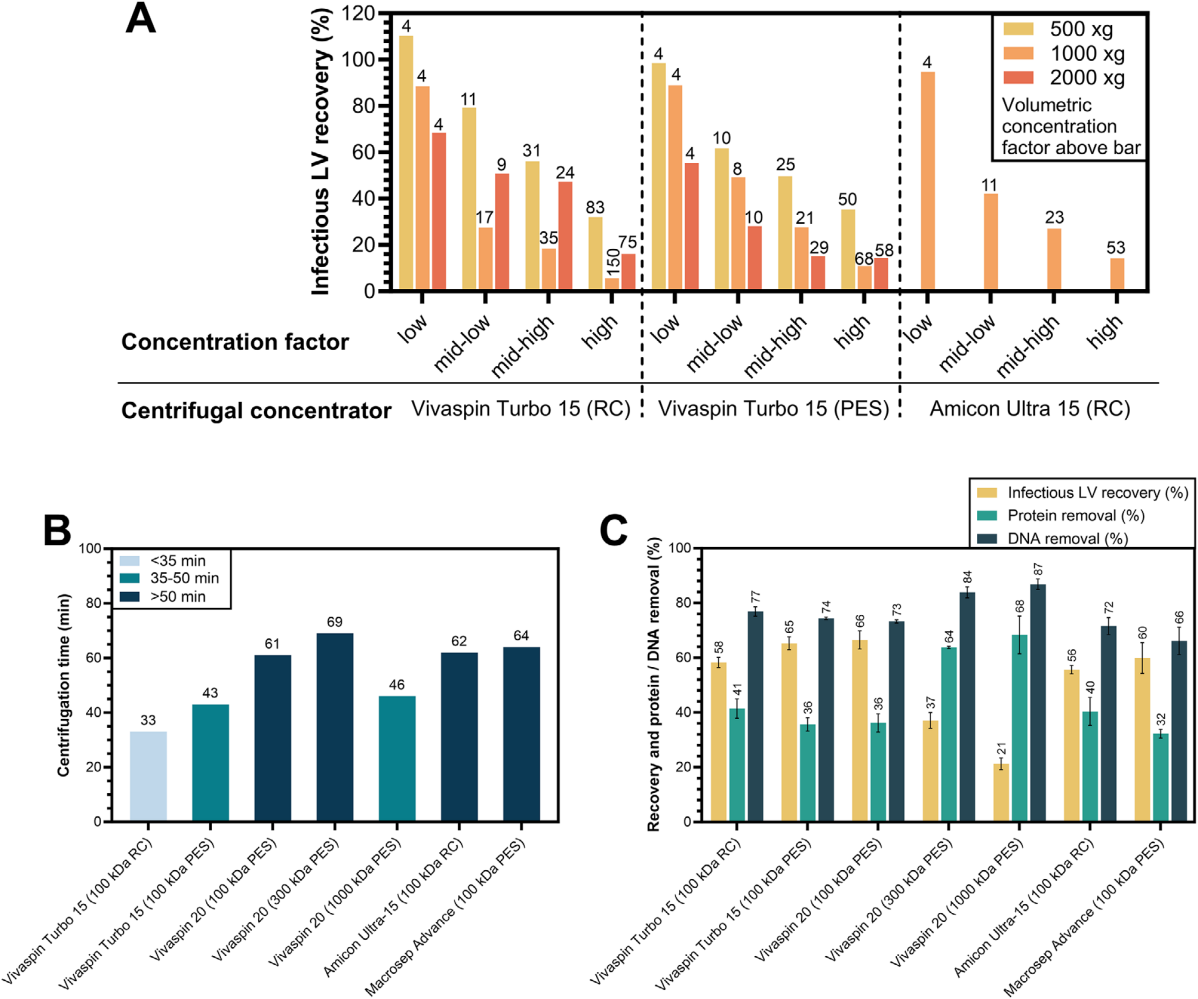
(A) Infectious LV recovery after UF at 500, 1000, and 2000 × *g* with Vivaspin Turbo 15 (RC and PES), and Amicon Ultra‐15 (RC), all having a MWCO of 100 kDa. The exact volumetric concentration factor is indicated above each bar, *n* = 1. (B) Centrifugation time for UF of LVs at 500 × *g* and (C) infectious LV recovery, protein removal and DNA removal after UF of LVs at 500 × *g* using different centrifugal ultrafilters. The LV samples from (B, C) were concentrated from 15 mL to approximately 2 mL (volumetric concentration factor = 7.5×). Values represent mean ± standard deviation, *n* = 3. LV, lentiviral vector; PES, polyethersulfone; RC, regenerated cellulose.

The first set of experiments evaluated process conditions for the concentrating LVs using commercial centrifugal ultrafilters. LV samples were concentrated to four different volumetric concentration factors, labeled above each bar in Figure 1A for clarity, as achieving consistent retentate volumes was difficult, particularly at high volumetric concentration factors. Vivaspin Turbo 15 with RC and PES membranes (100 kDa MWCO) were centrifuged at 500, 1000, and 2000 × *g*, respectively. Additionally, Amicon Ultra‐15 (100 kDa RC) was tested at 1000 × *g* to confirm observations across brands. Results showed that infectious LV recovery was highest at 500 × *g*, decreasing with higher centrifugal force and concentration factors. These findings were consistent among all tested centrifugal ultrafilters. Optimal process conditions for the concentration of LVs with centrifugal ultrafilters were identified at 500 × *g* with a volumetric concentration factor ≤10×, yielding 100% infectious LV recovery with a concentration factor of 4× and 60%–80% recovery at up to 11×. Given these favorable results, a concentration factor of ≤10× was optimal for further experiments, ensuring a balance between efficient concentration and maximizing LV recovery.

A comparison of seven centrifugal ultrafilters (comprising different membrane materials and MWCOs) from four suppliers was performed at the previously determined optimal process conditions (500 × *g*, and a 7.5× concentration factor). The centrifugation time needed to achieve this concentration factor and the infectious LV recovery, protein removal, and DNA removal are shown in Figure 1B, C, respectively. Although achieving high removal of impurities, the Vivaspin 20 with 300 and 1000 kDa PES was shown to be unsuitable for this application due to low LV recoveries. Taking a holistic view of the performance of the tested centrifugal ultrafilters with 100 kDa MWCO membranes, Vivaspin Turbo 15 (RC or PES), Vivaspin 20 (100 kDa PES), and Amicon Ultra‐15 showed the highest rates of infectious LV recovery (58%–66%) and DNA (66%–77%) and protein (32%–41%) removal. However, when also taking process time into account, the use of Vivaspin Turbo 15 is preferred, as the time to concentrate was 30%–48% less compared to the other three ultrafilters we tested with 100 kDa MWCO membranes.

#### 3.1.2 Comprehensive Evaluation of Membrane Materials for Optimized Lentiviral Concentration in Centrifugal Ultrafiltration

**FIGURE 2:**
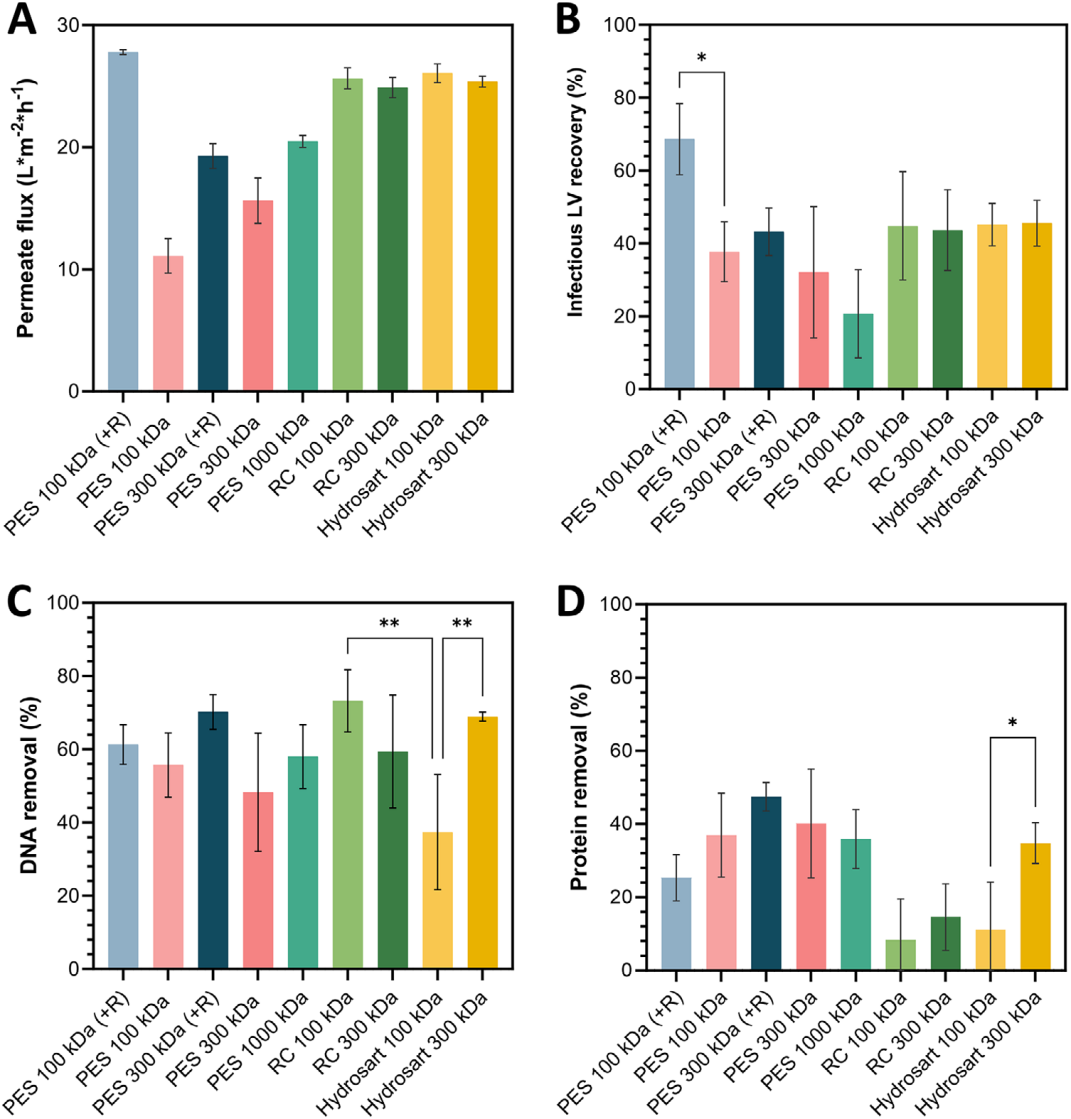
Centrifugal ultrafilter results. (A) Permeate flux (L m^−2^ h^−1^) during UF. (B) Infectious LV recoveries (%) of retentate samples. (C) DNA removal (%) and (D) Protein removal (%) in retentate samples, each depending on different membrane types. +R = fleece‐reinforced membrane. Data represent mean ± standard deviation for *n* = 4. LV, lentiviral vector; PES, polyethersulfone; RC, regenerated cellulose.

Commercially available Vivaspin ultrafilters in Figure 1 incorporate reinforced PES membranes with 100 and 300 kDa MWCO. To evaluate the impact of the membrane material on concentration using centrifugal ultrafilters, nine different membrane types were incorporated into custom‐made Vivaspin 15R devices. This allowed for a direct comparison of membranes independent of the concentrator design. Although Section 3.1.1 (Figure 1) compares commercial products, Section 3.1.2 (Figure 2) offers a comprehensive assessment of various membrane materials not necessarily available commercially in centrifugal ultrafilters. Based on prior findings, a centrifugal force of 500 × *g* and a concentration factor ≤10× was chosen for the experiments. Performance was evaluated based on permeate flux, infectious LV recovery, DNA, and protein removal (Figure 2A–D). The 100 kDa reinforced PES membrane yielded the fastest permeate flux with 27 L m^−1^ h^−2^ (LMH), closely followed by the cellulose‐based membranes (25 LMH). In contrast, nonreinforced PES membranes showed the lowest permeate flux (11 LMH). Similarly, the infectious LV recovery was highest (69%) with the reinforced 100 kDa PES membrane, aligning with the previous results in Figure 1C, which showed about 65% infectious LV recovery in commercial Vivaspin units with the same membrane. All cellulose‐based membranes performed similar with an average of 45% infectious LV recovery. Other membranes yielded significantly lower infectious LV recoveries, with the lowest being 21% for the 1000 kDa PES membrane; this sample was the only one to show an infectious recovery (8%) in the permeate (not shown).

Posconcentration, DNA removal ranged from 37% (100 kDa Hydrosart) to 70% (300 kDa reinforced PES), while protein removal varied from 8% (100 kDa RC) to 47% (300 kDa reinforced PES). Notably, Hydrosart RC was the only membrane showing a significant difference between MWCOs, as the impurity removal was significantly lower using a 100 kDa MWCO removed 37% DNA and 11% protein, whereas the 300 kDa MWCO removed 69% DNA and 34% protein.

All in all, the study found that 100 kDa reinforced PES membranes provided the highest infectious LV recovery (69%) and fastest permeate flux, making them the most effective, while 1000 kDa PES membranes had the lowest LV recovery and showed LV presence in the permeate.

### 3.2 Stirred Cells for Ultrafiltration—Rapid Screening Tool for Ultrafiltration Membranes

**FIGURE 3:**
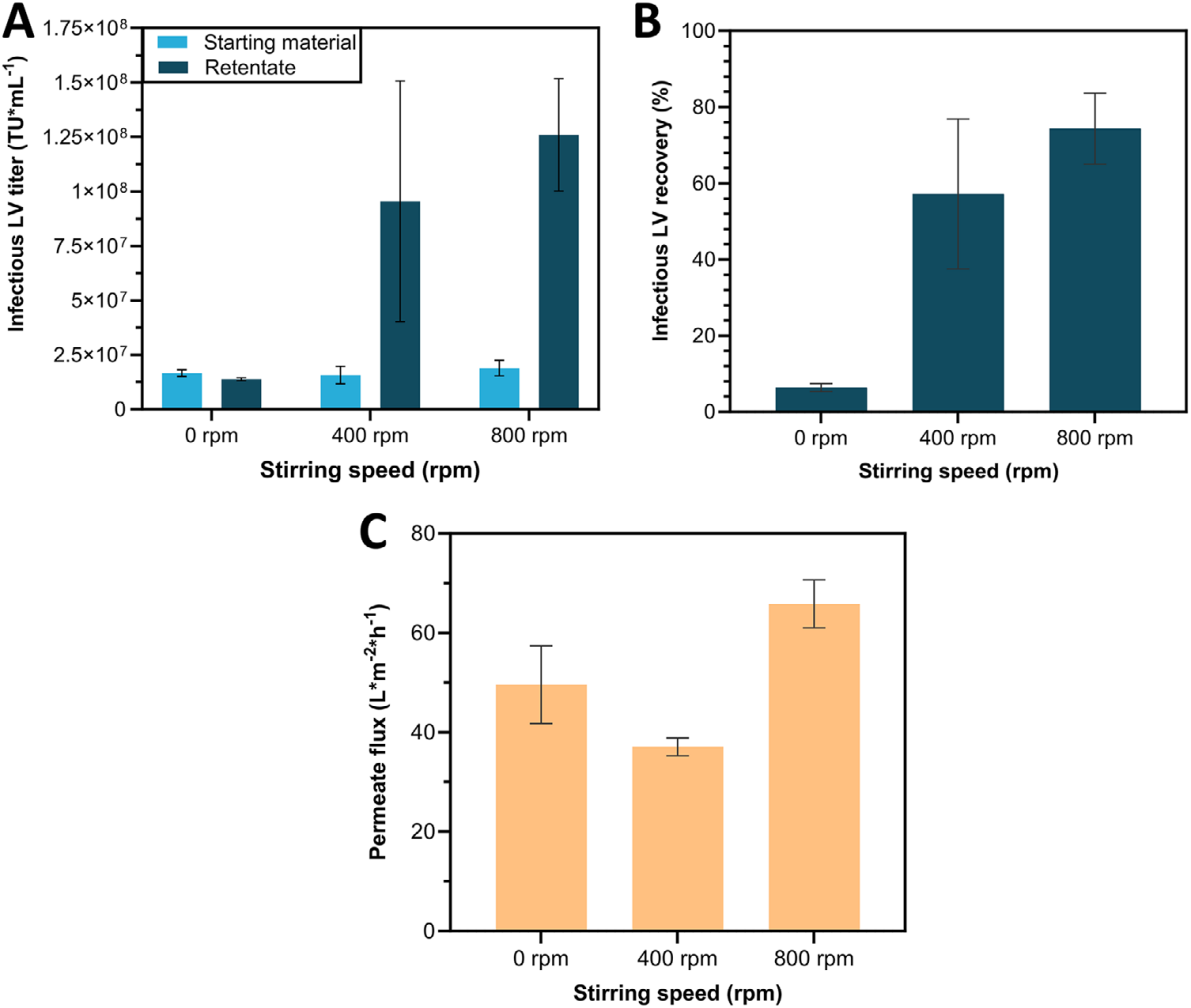
Influence of stirring speed on stirred cell ultrafiltration process. (A) Infectious lentiviral vector (LV) titers (TU mL^−1^) of starting material and retentate samples as well as (B) Infectious LV recovery (%) of retentate samples and (C) Permeate flux (L m^−2^ h^−1^) each varying depending on different stirring speeds. Data represent mean ± standard deviation for 0 rpm (*N* = 6) as well as 400 and 800 rpm (*N* = 4). LV, lentiviral vector.

Like centrifugal ultrafilters, stirred cells operate in dead‐end mode and are commonly used to characterize UF membranes due to easy membrane integration. In stirred cells, UF is pressure‐driven with air forcing fluid through a semipermeable membrane. The UF is stopped when the desired retentate volume is reached. Stirred cells with 10 mL capacity and 25 mm diameter membranes were tested at 2 bar, with stirring speeds of 0, 400, and 800 rpm using a reinforced 300 kDa PES membrane. The stirring speed was compared by reducing to the same retentate volume. Preliminary experiments (Figure 3) assessed the impact of turbulence flows from the integrated magnetic stirrer, revealing that the infectious LV recovery in retentate samples increased 9‐fold at 400 rpm and 11‐fold at 800 rpm, compared to no stirring. Additionally, permeate flow was highest with 66 LMH at 800 rpm. Based on these results, further experiments with a range of UF membranes (Table 1) were conducted at 800 rpm, as it showed the most promising outcome.

**FIGURE 4:**
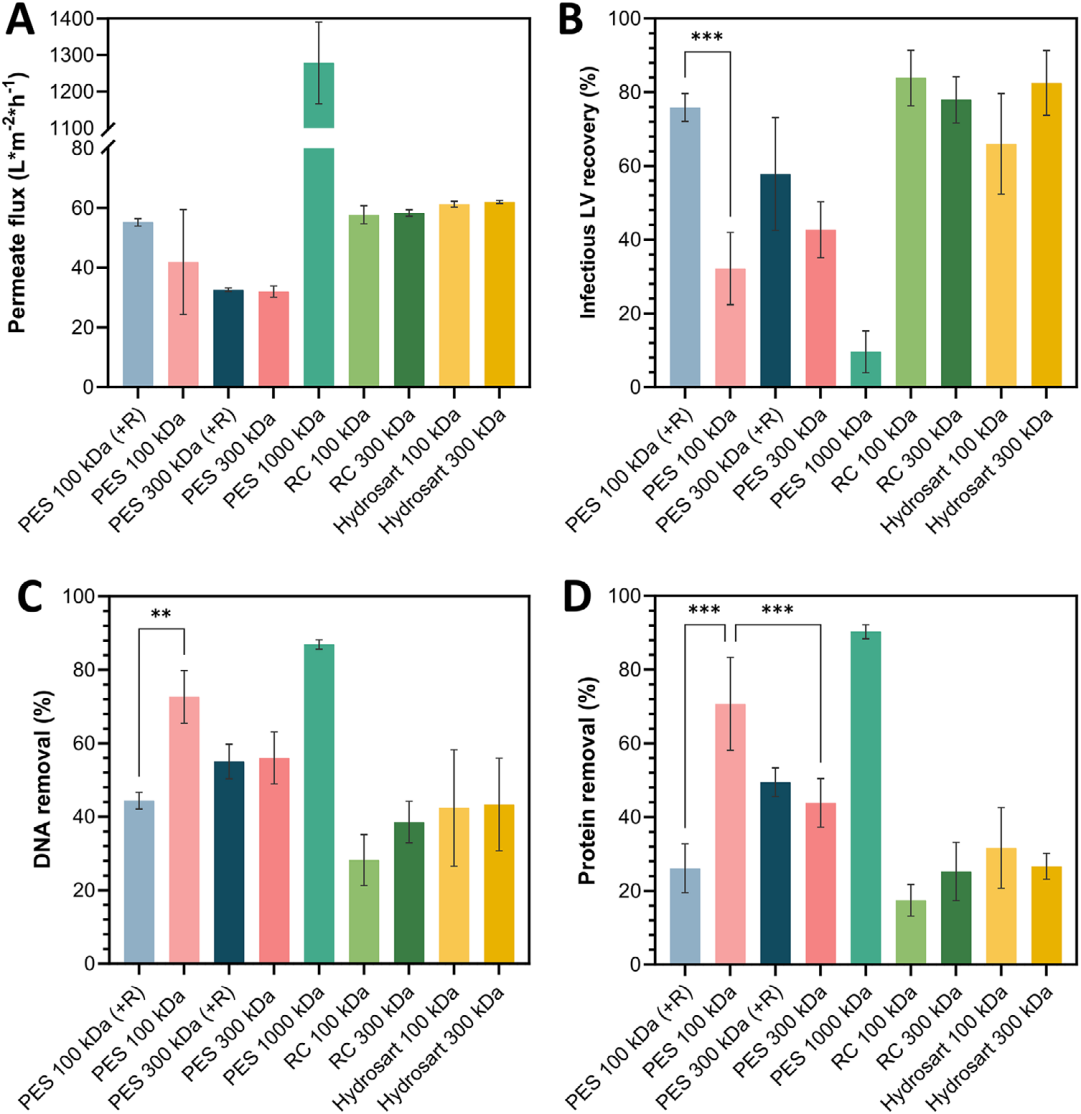
Stirred cell UF results. (A) Permeate flux (L m^−2^ h^−1^) during UF. (B) Infectious LV recoveries (%) of retentate samples. (C) DNA removal (%) and (D) Protein removal (%) in retentate samples, each depending on different membrane types. +R = fleece‐reinforced membrane. Data represent mean ± standard deviation for *n* = 4. LV, lentiviral vector; PES, polyethersulfone; RC, regenerated cellulose.

Permeate flux (Figure 4A) was highest with the 1000 kDa PES membrane at 1278 LMH. Membranes with lower MWCO showed significantly reduced permeate fluxes. Cellulose‐based membranes exhibited similar fluxes averaging 60 LMH, while fluxes varied among PES membranes by MWCO, with 100 kDa at 55 LMH (+R) and 41 LMH, and 300 kDa PES membranes averaged at 32 LMH.

Following UF, the highest infectious LV recoveries in retentate samples (Figure 4B) were obtained with cellulose‐based membranes ranging from 66% (100 kDa Hydrosart) to 83% (100 kDa RC and 300 kDa Hydrosart). PES membranes showed more variability with reinforced 100 kDa PES membranes yielding significantly higher infectious recoveries (76%) than nonreinforced counterparts (32%). This difference was not observed for 300 kDa PES membranes with reinforced versions yielding 58% infectious LV recovery and nonreinforced membranes 43%. The lowest infectious LV recovery was obtained with the 1000 kDa PES membrane (10%), which also showed an 8% LV recovery in the permeate (not shown).

Impurity removal varied by membrane type for dsDNA (Figure 4C) and protein (Figure 4D). The highest impurity removal was with the 1000 kDa PES membrane (≥87% for both DNA and protein), followed by the nonreinforced 100 kDa PES membrane with approximately 70%. Those retentate samples showed significantly higher removal rates compared to those obtained using a reinforced equivalent for DNA (44%) and protein (26%). The 300 kDa PES membranes removed about 55% of DNA and 44%–49% of protein. Cellulose‐based membranes generally showed lower impurity removal, with the 100 kDa RC membrane showing the least. The 300 kDa RC membrane equivalent enabled slightly higher removal rates with 38% (DNA) and 25% (protein). Hydrosart membranes showed similar impurity removal rates regardless of the MWCO, with about 43% DNA and about 30% protein removal.

The 1000 kDa PES membrane excelled in permeate flux and impurity removal but was poor in recovering infectious LVs. Reinforced 100 kDa PES provided a strong balance of LV recovery and impurity removal, outperforming its nonreinforced counterpart. Cellulose‐based membranes were strong in LV recovery but weaker in impurity removal, making them less effective for complete purification compared to PES membranes.

### 3.3 Crossflow Cassettes for Scalable Ultrafiltration—Screening With an Automated Multiparallel Small‐Scale Crossflow Filtration System

An automated Ambr Crossflow system was used to integrate a TFF device. In contrast to the other two devices, the retentate is recirculated in the system during concentration. Custom‐built filter cassettes, accommodating a mesh support layer on the permeate side and a single membrane layer (Table 1), were utilized. After flushing and equilibration, membranes were loaded with 40 mL of clarified LV solution and UF was performed with a TMP of 500 mbar and a flow rate of 10 mL min^−1^, reducing the retentate to a final volume of 6 mL.

**FIGURE 5:**
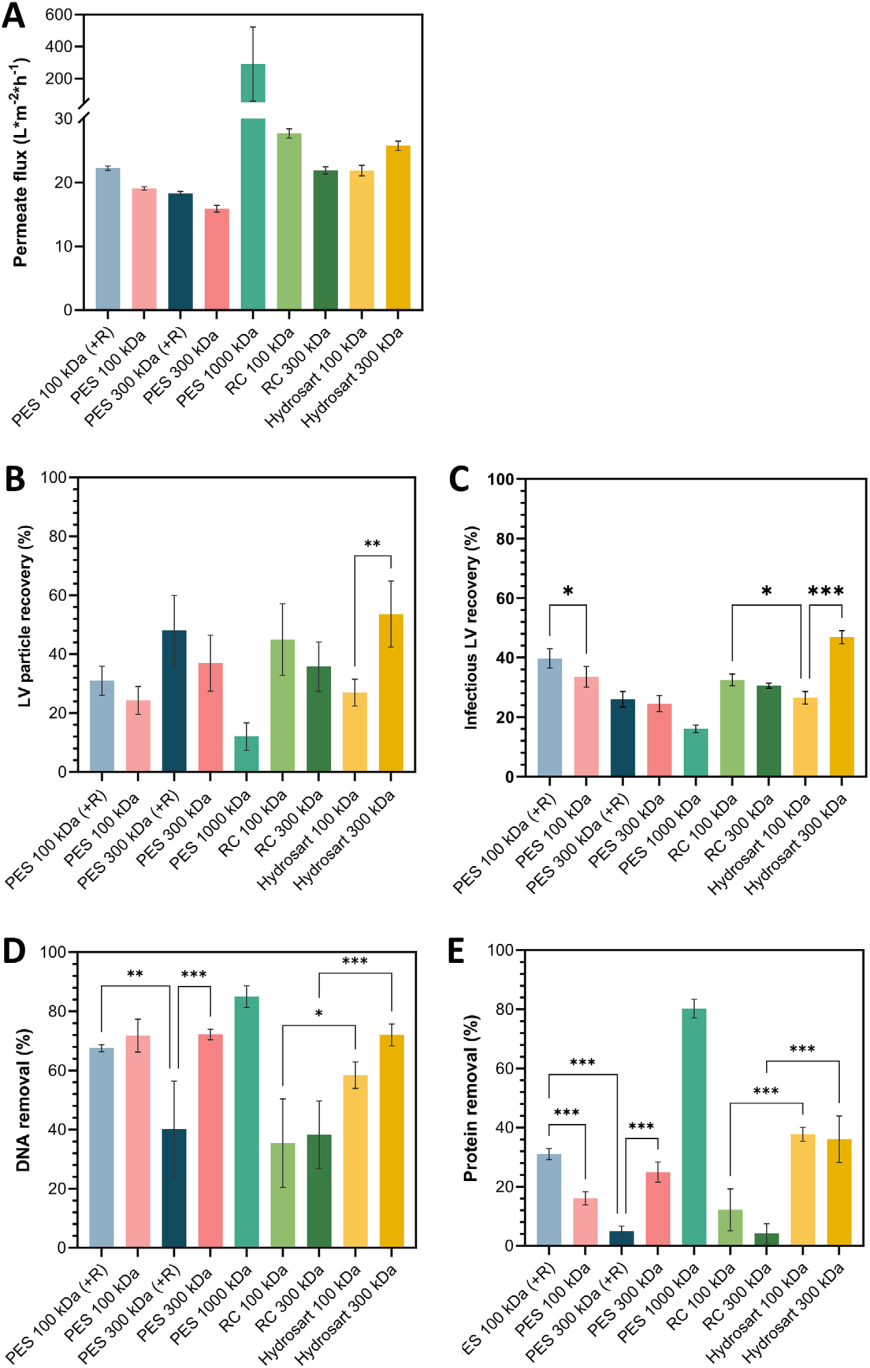
Crossflow cassettes UF experiments. (A) Permeate flux (L m^−2^ h^−1^) during UF (B) LV particle recovery (%) of retentate samples. (C) Infectious LV recoveries (%) of retentate samples. (D) DNA removal (%) and (E) Protein removal (%) in retentate samples, each depending on different membrane types. +R = fleece‐reinforced membrane. Data represent mean ± standard deviation for *n* = 4. LV, lentiviral vector; PES, polyethersulfone; RC, regenerated cellulose.

Permeate flux decreased over time during filtration (not shown), averaging around 18 LMH for the 100 and 300 kDa PES membranes and 23 LMH for cellulose‐based membranes (Figure 5A). The highest permeate flux of 290 LMH was achieved with the 1000 kDa PES membrane. The initial LV particle titer was 5.4 × 10^10^ VP mL^−1^. Analysis of LV particle and infectious recovery revealed higher LV particle recoveries overall (Figure 5B), with the 300 kDa Hydrosart membrane yielding the highest particle and infectious LV recoveries at 53% and 47%, respectively. The 100 kDa Hydrosart showed a significantly lower particle recovery at 27%, with the same trend mirrored in infectious LV recovery. In contrast, RC membranes exhibited slightly higher recoveries with the 100 kDa MWCO than with the 300 kDa membrane. The lowest LV recovery (≤16%) was obtained using the 1000 kDa PES membrane, while some infectious LVs were found in the permeate, which was not the case for all other membranes tested. However, this 1000 kDa PES membrane also achieved the highest impurity removal (Figure 5D, E), with 85% DNA and 80% protein removal, followed by the 100 kDa PES membranes, each at approximately 70% DNA removal. Notably, the reinforced 300 kDa membrane samples exhibited significantly lower DNA and protein removal rates with 38% and 12%, respectively. Cellulose‐based membranes showed consistent impurity removal rates across MWCOs of the same material; however, Hydrosart membranes achieved significantly higher impurity removals than RC.

## 4 Discussion

An ever‐increasing demand for LV therapies in the clinical landscape necessitates improving DSP methods. In this study, we investigated the optimization of LV UF by analyzing the impact of different filtration devices and membrane materials. We have used the same LV and membrane batch for all main experiments (only the stirring speed experiment with the stirring cell had another LV batch). This approach ensures that any observed effects can be attributed solely to the variations in membrane type or operation mode, effectively ruling out potential influences from differences in LV or membrane batches.

Optimal process conditions for UF of LVs using commercially available centrifugal ultrafilters from different suppliers were identified. We found that a centrifugal force of 500 × *g* and a concentration factor of ≤10 yielded the highest infectious LV recoveries. Higher *g* forces appear to introduce excessive shear stress, damaging LV particles and thus impairing their infectivity. Although specific data on shear stress effects on LV functionality in centrifugal ultrafilters are not yet published, shear stress is generally known to reduce LV infectivity and is a key issue in UF [8]. Our study is the first to examine infectious LV recovery across different *g* forces, and our findings indicate that increasing *g* force reduces LV recovery. This is likely due to shear stress, as centrifugation time did not affect recovery, as Figure 1 shows similar LV recoveries even with doubled centrifugation time in a cooled centrifuge, suggesting that time is not a significant factor. Moreover, at lower *g* forces longer centrifugation times were required, yet LV recovery was still better. A higher concentration factor may increase LV aggregation which can lower measured infectious titers as aggregates act as single infectious units [32]. We anticipate that particle titers measured by p24 ELISA would not show such as marked a decrease in recovery at high concentration factors; however, we could not confirm this due to limited sample volume. Comparing commercial centrifugal ultrafilters under the optimal process conditions, we found that those with larger MWCOs (300 and 1000 kDa) removed impurities most effectively but showed low LV recovery, potentially due LVs getting trapped in large membrane pores or experiencing greater shear stress. Among the 100 kDa centrifugal ultrafilters, the highest LV recovery, impurity removal, and the shortest process time were achieved with the Vivaspin Turbo 15 PES.

Optimized parameters were used to screen different membrane materials in centrifugal ultrafilters, enabling direct membrane comparisons independent of the concentrator design. The same membrane materials were also tested in stirred cells and crossflow cassettes. Preliminary experiments identified optimal process conditions for stirred cells at 800 rpm and a pressure of 2 bar, while crossflow cassette conditions (e.g., TMP) relied on literature data [26, 27, 28, 29]. Across devices, a 100 kDa reinforced PES membrane consistently yielded some of the highest infectious LV recoveries in retentate samples, significantly outperforming its non‐reinforced counterpart. This difference likely results from the structure of the reinforced membrane, which uses polymer fleece lining to withstand higher pressures and have tighter pore sizes. These design elements effectively impede LV passage while maintaining sufficient permeate flux. Larger pore sizes appear to increase shear stress, leading to a destruction of LV particles and thus lower infectious LV titers as observed for higher MWCO PES membranes. In contrast, cellulose‐based membranes showed no MWCO effect on infectious LV recovery, with similar LV recovery rates, especially in the stirred cells and centrifugal ultrafilters.

Our findings highlight the impact of filtration device choice on the efficiency of LV recovery, with the stirred cell consistently outperforming the centrifugal ultrafilters and crossflow cassettes. Factors such as operating principles, loading density, concentration polarization, and gel‐layer formation contribute to these differences. Permeate flux evaluation was highest in stirred cells and similar across membranes in crossflow cassettes and centrifugal ultrafilters. This can be attributed to the lower loading density in the stirred cell (∼26 vs. ∼38 L m^−2^ in other devices), which is related to the device designs. Moreover, the applied additional stirring in the stirred cells reduces cake‐layer formation and thus concentration polarization and gel‐layer formation. Permeate flux is further improved by higher applied pressure, as it reduces the osmotic counter pressure that arises with higher concentration polarization. It can therefore be argued that infectious LV recovery was impaired in the centrifugal ultrafilters and crossflow cassettes by increased LV agglomeration due to the increased loading density (38 vs. 26 L m^−2^ in the stirred cell) and decreased permeate flux leading to more concentration polarization. Despite similar loading density and permeate fluxes for each membrane, infectious LV recoveries were for the majority of the membrane materials 1.3‐ to 1.7‐fold higher in centrifugal ultrafilters than in crossflow cassettes. Literature supports the use of reinforced 100 kDa PES membranes in centrifugal ultrafilters, achieving the highest infectious LV recovery, although only a limited membrane comparison was performed [33, 34]. Using crossflow cassettes the LV recovery remained ≤47% underscoring the need for process optimization, particularly a TMP scouting as done by Mendes et al. [7]. This would require a TMP scouting for every membrane at different flow rates to identify the optimal TMP and flow rate combination. Adjusting moreover, flow rate and TMP may improve LV recovery by reducing the filtration time and LV recirculation time in the system, thereby potentially reducing mechanical stress. While TFF for LV concentration has shown considerably high LV recoveries of 70%–100% in other studies [27, 29], our reliance on literature parameters due to time and material constraints likely limited yields in this study.

Our investigation into the effects of membrane material and filtration device on impurity removal suggests a stronger interplay between these parameters than observed for infectious LV recovery. Generally, membranes with higher MWCOs are assumed to be more effective at removing impurities such as DNA and protein. However, significant differences in impurity removal between different MWCO of the same membrane material were only observed for UF with Hydrosart membranes in centrifugal ultrafilters. Other than that, higher MWCO of the same membrane material did not display higher impurity removal, except, with a 1000 kDa cutoff, which achieved >80% impurity removal in stirred cells or crossflow cassettes. Nonetheless, this MWCO had also the lowest infectious LV recoveries with infectious LVs detected in the permeate, rendering this MWCO unsuitable for LV retention. That aside, a DNA removal of about 72% was achieved using different membrane types and device combinations, for example, a 300 kDa Hydrosart or 100 and 300 kDa non‐reinforced PES membrane in crossflow cassettes. In contrast, protein removal was generally <50%, possibly due to membrane–protein and protein–protein interactions, the larger size and higher concentrations of proteins compared to DNA (especially after DNase treatment) may hinder protein passage through the membrane. Maintaining LV particle integrity and removing impurities during the preceding steps (e.g., clarification), positively impacts UF performance, as cleaner input material reduces the burden on the UF filter. Furthermore, preserving LV particle integrity during UF minimizes the formation of fragmented particles, which would otherwise increase protein content, which would put more burden on the filter.

Efficient LV production is crucial to meet the growing demand for LV‐based therapeutics. An essential aspect of the production process is optimizing UF techniques within the DSP, which was investigated in this study by screening different membrane types and UF devices. The operation of the centrifugal ultrafilters and the stirred cell were optimized beforehand, resulting in good LV recoveries of up to 84%, with the stirred cell generally outperforming the other devices. Time and material constraints led to the selection of TFF parameters based on literature, resulting in poorer yields, which could be improved with further optimization. Overall, the reinforced 100 kDa PES and a 300 kDa Hydrosart membrane performed best for LV recovery and impurity removal. Notably, protein removal was more challenging compared to DNA and a higher MWCO did not consistently enhance the removal of impurities. Although exploration of a higher MWCO membrane (1000 kDa) showed promising results for impurity removal, it failed to retain LVs effectively.

We conclude that UF optimization should involve testing different membrane materials and MWCOs for a specific target. Further, the performance of a membrane can vary across devices, thus process parameters should be individually optimized. MWCO values are not universally consistent due to pore size variations depending on the membrane material and manufacturer, even when the same MWCO specification is indicated. It is therefore always advisable to screen the available membranes thoroughly.

## Author Contributions

**Jennifer J. Labisch:** conceptualization, methodology, investigation, formal analysis, visualization, writing—original draft. **Maria Evangelopoulou:** investigation, formal analysis, visualization, writing—original draft. **Tobias Schleuß:** conceptualization, supervision, writing—review and editing. **Andreas Pickl:** supervision, project administration, writing—review and editing.

## Conflicts of Interest

The authors declare no conflicts of interest.

## Supporting information



Supporting Information

## Data Availability

The data that support the findings of this study are available from the corresponding author upon reasonable request.
