# Supplementary material for: Investigating Ultrafiltration Membranes and Operation Modes for Improved Lentiviral Vector Processing
Source: Eng Life Sci. 2025 Jan 3;25(1):e202400057. doi: 10.1002/elsc.202400057 (PMC11717145; doi:10.1002/elsc.202400057)
Supplement: Supplementary file 1 — Supporting Information [file ELSC-25-e202400057-s001.docx]

**Supplementary Material**


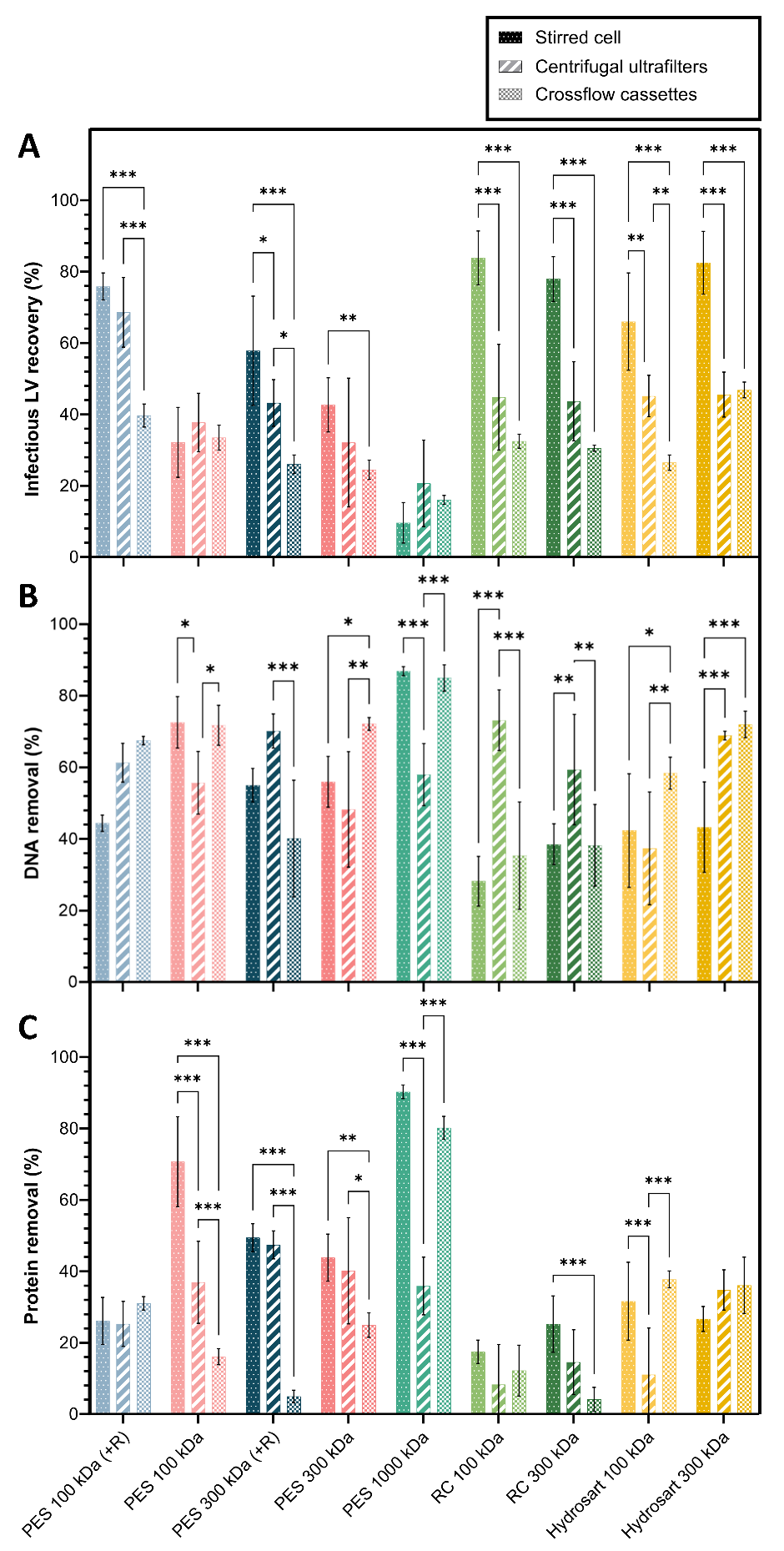


**Figure S1: (A) Infectious LV recovery, (B) DNA removal, (C) protein removal of retentate samples after UF with nine different membranes using the three different devices.** Type A = fleece-reinforced membrane. type B = no fleece-reinforcement. Data represent mean ± standard deviation for N = 4. Statistical significances are shown with p-values indicating: * p ≤ 0.05. ** p ≤ 0.01. *** p < 0.001.

**Table S1:** Tukey’s multiple comparison test of infectious LV recovery in retentate samples using different commercially available centrifugal concentrators. Abbreviations: CI – confidence interval, PES – polyethersulfone, RC – regenerated cellulose.

| **Membrane Types** | **Mean Diff.** | **95.00% CI of diff.** | **Summary** | **Adjusted p Value** |
| --- | --- | --- | --- | --- |
| Vivaspin Turbo 15 (100 kDa RC) vs. Vivaspin Turbo 15 (100 kDa PES) | -7.04 | -15.19 to 1.112 | ns | 0.131 |
| Vivaspin Turbo 15 (100 kDa RC) vs. Vivaspin 20 (100 kDa PES) | -8.23 | -16.38 to -0.07790 | * | 0.047 |
| Vivaspin Turbo 15 (100 kDa RC) vs. Vivaspin 20 (300 kDa PES) | 21.19 | 13.04 to 29.34 | *** | <.001 |
| Vivaspin Turbo 15 (100 kDa RC) vs. Vivaspin 20 (1000 kDa PES) | 37.03 | 28.88 to 45.18 | *** | <.001 |
| Vivaspin Turbo 15 (100 kDa RC) vs. Amicon Ultra-15 (100 kDa RC) | 2.62 | -5.532 to 10.77 | ns | 0.952 |
| Vivaspin Turbo 15 (100 kDa RC) vs. Macrosep Advance (100 kDa PES) | -1.64 | -9.792 to 6.512 | ns | 0.996 |
| Vivaspin Turbo 15 (100 kDa PES) vs. Vivaspin 20 (100 kDa PES) | -1.19 | -9.342 to 6.962 | ns | >.999 |
| Vivaspin Turbo 15 (100 kDa PES) vs. Vivaspin 20 (300 kDa PES) | 28.23 | 20.08 to 36.38 | *** | <.001 |
| Vivaspin Turbo 15 (100 kDa PES) vs. Vivaspin 20 (1000 kDa PES) | 44.07 | 35.92 to 52.22 | *** | <.001 |
| Vivaspin Turbo 15 (100 kDa PES) vs. Amicon Ultra-15 (100 kDa RC) | 9.66 | 1.508 to 17.81 | * | 0.011 |
| Vivaspin Turbo 15 (100 kDa PES) vs. Macrosep Advance (100 kDa PES) | 5.4 | -2.752 to 13.55 | ns | 0.4 |
| Vivaspin 20 (100 kDa PES) vs. Vivaspin 20 (300 kDa PES) | 29.42 | 21.27 to 37.57 | *** | <.001 |
| Vivaspin 20 (100 kDa PES) vs. Vivaspin 20 (1000 kDa PES) | 45.26 | 37.11 to 53.41 | *** | <.001 |
| Vivaspin 20 (100 kDa PES) vs. Amicon Ultra-15 (100 kDa RC) | 10.85 | 2.698 to 19.00 | ** | 0.003 |
| Vivaspin 20 (100 kDa PES) vs. Macrosep Advance (100 kDa PES) | 6.59 | -1.562 to 14.74 | ns | 0.185 |
| Vivaspin 20 (300 kDa PES) vs. Vivaspin 20 (1000 kDa PES) | 15.84 | 7.688 to 23.99 | *** | <.001 |
| Vivaspin 20 (300 kDa PES) vs. Amicon Ultra-15 (100 kDa RC) | -18.57 | -26.72 to -10.42 | *** | <.001 |
| Vivaspin 20 (300 kDa PES) vs. Macrosep Advance (100 kDa PES) | -22.83 | -30.98 to -14.68 | *** | <.001 |
| Vivaspin 20 (1000 kDa PES) vs. Amicon Ultra-15 (100 kDa RC) | -34.41 | -42.56 to -26.26 | *** | <.001 |
| Vivaspin 20 (1000 kDa PES) vs. Macrosep Advance (100 kDa PES) | -38.67 | -46.82 to -30.52 | *** | <.001 |
| Amicon Ultra-15 (100 kDa RC) vs. Macrosep Advance (100 kDa PES) | -4.26 | -12.41 to 3.892 | ns | 0.672 |

**Table S2:** Tukey’s multiple comparison test of protein removal in retentate samples using different commercially available centrifugal concentrators. Abbreviations: CI – confidence interval, PES – polyethersulfone, RC – regenerated cellulose.

| **Membrane Types** | **Mean Diff.** | **95.00% CI of diff.** | **Summary** | **Adjusted p Value** |
| --- | --- | --- | --- | --- |
| Vivaspin Turbo 15 (100 kDa RC) vs. Vivaspin Turbo 15 (100 kDa PES) | 5.76 | -2.392 to 13.91 | ns | 0.324 |
| Vivaspin Turbo 15 (100 kDa RC) vs. Vivaspin 20 (100 kDa PES) | 5.19 | -2.962 to 13.34 | ns | 0.448 |
| Vivaspin Turbo 15 (100 kDa RC) vs. Vivaspin 20 (300 kDa PES) | -22.36 | -30.51 to -14.21 | *** | <.001 |
| Vivaspin Turbo 15 (100 kDa RC) vs. Vivaspin 20 (1000 kDa PES) | -26.95 | -35.10 to -18.80 | *** | <.001 |
| Vivaspin Turbo 15 (100 kDa RC) vs. Amicon Ultra-15 (100 kDa RC) | 1 | -7.152 to 9.152 | ns | >.999 |
| Vivaspin Turbo 15 (100 kDa RC) vs. Macrosep Advance (100 kDa PES) | 9.14 | 0.9879 to 17.29 | * | 0.019 |
| Vivaspin Turbo 15 (100 kDa PES) vs. Vivaspin 20 (100 kDa PES) | -0.57 | -8.722 to 7.582 | ns | >.999 |
| Vivaspin Turbo 15 (100 kDa PES) vs. Vivaspin 20 (300 kDa PES) | -28.12 | -36.27 to -19.97 | *** | <.001 |
| Vivaspin Turbo 15 (100 kDa PES) vs. Vivaspin 20 (1000 kDa PES) | -32.71 | -40.86 to -24.56 | *** | <.001 |
| Vivaspin Turbo 15 (100 kDa PES) vs. Amicon Ultra-15 (100 kDa RC) | -4.76 | -12.91 to 3.392 | ns | 0.551 |
| Vivaspin Turbo 15 (100 kDa PES) vs. Macrosep Advance (100 kDa PES) | 3.38 | -4.772 to 11.53 | ns | 0.856 |
| Vivaspin 20 (100 kDa PES) vs. Vivaspin 20 (300 kDa PES) | -27.55 | -35.70 to -19.40 | *** | <.001 |
| Vivaspin 20 (100 kDa PES) vs. Vivaspin 20 (1000 kDa PES) | -32.14 | -40.29 to -23.99 | *** | <.001 |
| Vivaspin 20 (100 kDa PES) vs. Amicon Ultra-15 (100 kDa RC) | -4.19 | -12.34 to 3.962 | ns | 0.689 |
| Vivaspin 20 (100 kDa PES) vs. Macrosep Advance (100 kDa PES) | 3.95 | -4.202 to 12.10 | ns | 0.743 |
| Vivaspin 20 (300 kDa PES) vs. Vivaspin 20 (1000 kDa PES) | -4.59 | -12.74 to 3.562 | ns | 0.592 |
| Vivaspin 20 (300 kDa PES) vs. Amicon Ultra-15 (100 kDa RC) | 23.36 | 15.21 to 31.51 | *** | <.001 |
| Vivaspin 20 (300 kDa PES) vs. Macrosep Advance (100 kDa PES) | 31.5 | 23.35 to 39.65 | *** | <.001 |
| Vivaspin 20 (1000 kDa PES) vs. Amicon Ultra-15 (100 kDa RC) | 27.95 | 19.80 to 36.10 | *** | <.001 |
| Vivaspin 20 (1000 kDa PES) vs. Macrosep Advance (100 kDa PES) | 36.09 | 27.94 to 44.24 | *** | <.001 |
| Amicon Ultra-15 (100 kDa RC) vs. Macrosep Advance (100 kDa PES) | 8.14 | -0.01210 to 16.29 | ns | 0.051 |

**Table S3:** Tukey’s multiple comparison test of DNA removal in retentate samples using different commercially available centrifugal concentrators. Abbreviations: CI – confidence interval, PES – polyethersulfone, RC – regenerated cellulose.

| **Membrane Types** | **Mean Diff.** | **95.00% CI of diff.** | **Summary** | **Adjusted p Value** |
| --- | --- | --- | --- | --- |
| Vivaspin Turbo 15 (100 kDa RC) vs. Vivaspin Turbo 15 (100 kDa PES) | 2.49 | -5.662 to 10.64 | ns | 0.963 |
| Vivaspin Turbo 15 (100 kDa RC) vs. Vivaspin 20 (100 kDa PES) | 3.61 | -4.542 to 11.76 | ns | 0.814 |
| Vivaspin Turbo 15 (100 kDa RC) vs. Vivaspin 20 (300 kDa PES) | -6.99 | -15.14 to 1.162 | ns | 0.136 |
| Vivaspin Turbo 15 (100 kDa RC) vs. Vivaspin 20 (1000 kDa PES) | -9.99 | -18.14 to -1.838 | ** | 0.008 |
| Vivaspin Turbo 15 (100 kDa RC) vs. Amicon Ultra-15 (100 kDa RC) | 5.33 | -2.822 to 13.48 | ns | 0.416 |
| Vivaspin Turbo 15 (100 kDa RC) vs. Macrosep Advance (100 kDa PES) | 10.75 | 2.598 to 18.90 | ** | 0.003 |
| Vivaspin Turbo 15 (100 kDa PES) vs. Vivaspin 20 (100 kDa PES) | 1.12 | -7.032 to 9.272 | ns | >.999 |
| Vivaspin Turbo 15 (100 kDa PES) vs. Vivaspin 20 (300 kDa PES) | -9.48 | -17.63 to -1.328 | * | 0.014 |
| Vivaspin Turbo 15 (100 kDa PES) vs. Vivaspin 20 (1000 kDa PES) | -12.48 | -20.63 to -4.328 | *** | <.001 |
| Vivaspin Turbo 15 (100 kDa PES) vs. Amicon Ultra-15 (100 kDa RC) | 2.84 | -5.312 to 10.99 | ns | 0.931 |
| Vivaspin Turbo 15 (100 kDa PES) vs. Macrosep Advance (100 kDa PES) | 8.26 | 0.1079 to 16.41 | * | 0.045 |
| Vivaspin 20 (100 kDa PES) vs. Vivaspin 20 (300 kDa PES) | -10.6 | -18.75 to -2.448 | ** | 0.004 |
| Vivaspin 20 (100 kDa PES) vs. Vivaspin 20 (1000 kDa PES) | -13.6 | -21.75 to -5.448 | *** | <.001 |
| Vivaspin 20 (100 kDa PES) vs. Amicon Ultra-15 (100 kDa RC) | 1.72 | -6.432 to 9.872 | ns | 0.994 |
| Vivaspin 20 (100 kDa PES) vs. Macrosep Advance (100 kDa PES) | 7.14 | -1.012 to 15.29 | ns | 0.121 |
| Vivaspin 20 (300 kDa PES) vs. Vivaspin 20 (1000 kDa PES) | -3 | -11.15 to 5.152 | ns | 0.912 |
| Vivaspin 20 (300 kDa PES) vs. Amicon Ultra-15 (100 kDa RC) | 12.32 | 4.168 to 20.47 | *** | <.001 |
| Vivaspin 20 (300 kDa PES) vs. Macrosep Advance (100 kDa PES) | 17.74 | 9.588 to 25.89 | *** | <.001 |
| Vivaspin 20 (1000 kDa PES) vs. Amicon Ultra-15 (100 kDa RC) | 15.32 | 7.168 to 23.47 | *** | <.001 |
| Vivaspin 20 (1000 kDa PES) vs. Macrosep Advance (100 kDa PES) | 20.74 | 12.59 to 28.89 | *** | <.001 |
| Amicon Ultra-15 (100 kDa RC) vs. Macrosep Advance (100 kDa PES) | 5.42 | -2.732 to 13.57 | ns | 0.395 |

**Table S4:** Tukey’s multiple comparison test of infectious LV recovery in retentate samples using different membrane types in a centrifugal ultrafilter. Abbreviations: CI – confidence interval, HY – Hydrosart, PES – polyethersulfone, RC – regenerated cellulose.

| **Membrane types** | **Mean Diff.** | **95% CI of diff.** | **Summary** | **Adjusted p Value** |
| --- | --- | --- | --- | --- |
| PES 100 kDa (+R) vs. PES 100 kDa | 30.89 | 4.662 to 57.12 | * | 0.012 |
| PES 100 kDa (+R) vs. PES 300 kDa (+R) | 25.40 | -0.8282 to 51.63 | ns | 0.063 |
| PES 100 kDa (+R) vs. PES 300 kDa | 36.51 | 10.28 to 62.74 | ** | 0.002 |
| PES 100 kDa (+R) vs. PES 1000 kDa | 47.93 | 21.70 to 74.16 | *** | <0.001 |
| PES 100 kDa (+R) vs. RC 100 kDa | 23.80 | -2.428 to 50.03 | ns | 0.098 |
| PES 100 kDa (+R) vs. RC 300 kDa | 24.97 | -1.258 to 51.20 | ns | 0.071 |
| PES 100 kDa (+R) vs. HY 100 kDa | 23.47 | -2.758 to 49.70 | ns | 0.107 |
| PES 100 kDa (+R) vs. HY 300 kDa | 23.06 | -3.168 to 49.29 | ns | 0.119 |
| PES 100 kDa vs. PES 300 kDa (+R) | -5.49 | -31.72 to 20.74 | ns | 0.998 |
| PES 100 kDa vs. PES 300 kDa | 5.62 | -20.61 to 31.85 | ns | 0.998 |
| PES 100 kDa vs. PES 1000 kDa | 17.04 | -9.188 to 43.27 | ns | 0.442 |
| PES 100 kDa vs. RC 100 kDa | -7.09 | -33.32 to 19.14 | ns | 0.990 |
| PES 100 kDa vs. RC 300 kDa | -5.92 | -32.15 to 20.31 | ns | 0.997 |
| PES 100 kDa vs. HY 100 kDa | -7.42 | -33.65 to 18.81 | ns | 0.987 |
| PES 100 kDa vs. HY 300 kDa | -7.83 | -34.06 to 18.40 | ns | 0.982 |
| PES 300 kDa (+R) vs. PES 300 kDa | 11.11 | -15.12 to 37.34 | ns | 0.878 |
| PES 300 kDa (+R) vs. PES 1000 kDa | 22.53 | -3.698 to 48.76 | ns | 0.136 |
| PES 300 kDa (+R) vs. RC 100 kDa | -1.60 | -27.83 to 24.63 | ns | >0.999 |
| PES 300 kDa (+R) vs. RC 300 kDa | -0.430 | -26.66 to 25.80 | ns | >0.999 |
| PES 300 kDa (+R) vs. HY 100 kDa | -1.93 | -28.16 to 24.30 | ns | >0.999 |
| PES 300 kDa (+R) vs. HY 300 kDa | -2.34 | -28.57 to 23.89 | ns | >0.999 |
| PES 300 kDa vs. PES 1000 kDa | 11.42 | -14.81 to 37.65 | ns | 0.862 |
| PES 300 kDa vs. RC 100 kDa | -12.71 | -38.94 to 13.52 | ns | 0.780 |
| PES 300 kDa vs. RC 300 kDa | -11.54 | -37.77 to 14.69 | ns | 0.855 |
| PES 300 kDa vs. HY 100 kDa | -13.04 | -39.27 to 13.19 | ns | 0.757 |
| PES 300 kDa vs. HY 300 kDa | -13.45 | -39.68 to 12.78 | ns | 0.727 |
| PES 1000 kDa vs. RC 100 kDa | -24.13 | -50.36 to 2.098 | ns | 0.090 |
| PES 1000 kDa vs. RC 300 kDa | -22.96 | -49.19 to 3.268 | ns | 0.122 |
| PES 1000 kDa vs. HY 100 kDa | -24.46 | -50.69 to 1.768 | ns | 0.082 |
| PES 1000 kDa vs. HY 300 kDa | -24.87 | -51.10 to 1.358 | ns | 0.073 |
| RC 100 kDa vs. RC 300 kDa | 1.17 | -25.06 to 27.40 | ns | >0.999 |
| RC 100 kDa vs. HY 100 kDa | -0.330 | -26.56 to 25.90 | ns | >0.999 |
| RC 100 kDa vs. HY 300 kDa | -0.740 | -26.97 to 25.49 | ns | >0.999 |
| RC 300 kDa vs. HY 100 kDa | -1.50 | -27.73 to 24.73 | ns | >0.999 |
| RC 300 kDa vs. HY 300 kDa | -1.91 | -28.14 to 24.32 | ns | >0.999 |
| HY 100 kDa vs. HY 300 kDa | -0.410 | -26.64 to 25.82 | ns | >0.999 |

**Table S5:** Tukey’s multiple comparison test of protein removal in retentate samples using different membrane types in a centrifugal ultrafilter. Abbreviations: CI – confidence interval, HY – Hydrosart, PES – polyethersulfone, RC – regenerated cellulose.

| **Membrane types** | **Mean Diff.** | **95% CI of diff.** | **Summary** | **Adjusted p Value** |
| --- | --- | --- | --- | --- |
| PES 100 kDa (+R) vs. PES 100 kDa | -11.65 | -35.24 to 11.94 | ns | 0.76 |
| PES 100 kDa (+R) vs. PES 300 kDa (+R) | -22.14 | -45.73 to 1.454 | ns | 0.08 |
| PES 100 kDa (+R) vs. PES 300 kDa | -14.87 | -38.46 to 8.724 | ns | 0.48 |
| PES 100 kDa (+R) vs. PES 1000 kDa | -10.60 | -34.19 to 12.99 | ns | 0.84 |
| PES 100 kDa (+R) vs. RC 100 kDa | 17.00 | -6.594 to 40.59 | ns | 0.31 |
| PES 100 kDa (+R) vs. RC 300 kDa | 10.73 | -12.86 to 34.32 | ns | 0.83 |
| PES 100 kDa (+R) vs. HY 100 kDa | 14.26 | -9.334 to 37.85 | ns | 0.54 |
| PES 100 kDa (+R) vs. HY 300 kDa | -9.50 | -33.09 to 14.09 | ns | 0.90 |
| PES 100 kDa vs. PES 300 kDa (+R) | -10.49 | -34.08 to 13.10 | ns | 0.85 |
| PES 100 kDa vs. PES 300 kDa | -3.22 | -26.81 to 20.37 | ns | >0.99 |
| PES 100 kDa vs. PES 1000 kDa | 1.05 | -22.54 to 24.64 | ns | >0.99 |
| PES 100 kDa vs. RC 100 kDa | 28.65 | 5.056 to 52.24 | ** | 0.009 |
| PES 100 kDa vs. RC 300 kDa | 22.38 | -1.214 to 45.97 | ns | 0.07 |
| PES 100 kDa vs. HY 100 kDa | 25.91 | 2.316 to 49.50 | * | 0.02 |
| PES 100 kDa vs. HY 300 kDa | 2.15 | -21.44 to 25.74 | ns | >0.99 |
| PES 300 kDa (+R) vs. PES 300 kDa | 7.27 | -16.32 to 30.86 | ns | 0.98 |
| PES 300 kDa (+R) vs. PES 1000 kDa | 11.54 | -12.05 to 35.13 | ns | 0.77 |
| PES 300 kDa (+R) vs. RC 100 kDa | 39.14 | 15.55 to 62.73 | *** | <0.001 |
| PES 300 kDa (+R) vs. RC 300 kDa | 32.87 | 9.276 to 56.46 | ** | 0.002 |
| PES 300 kDa (+R) vs. HY 100 kDa | 36.40 | 12.81 to 59.99 | *** | <0.001 |
| PES 300 kDa (+R) vs. HY 300 kDa | 12.64 | -10.95 to 36.23 | ns | 0.68 |
| PES 300 kDa vs. PES 1000 kDa | 4.27 | -19.32 to 27.86 | ns | >0.99 |
| PES 300 kDa vs. RC 100 kDa | 31.87 | 8.276 to 55.46 | ** | 0.003 |
| PES 300 kDa vs. RC 300 kDa | 25.60 | 2.006 to 49.19 | * | 0.03 |
| PES 300 kDa vs. HY 100 kDa | 29.13 | 5.536 to 52.72 | ** | 0.008 |
| PES 300 kDa vs. HY 300 kDa | 5.37 | -18.22 to 28.96 | ns | >0.99 |
| PES 1000 kDa vs. RC 100 kDa | 27.60 | 4.006 to 51.19 | * | 0.01 |
| PES 1000 kDa vs. RC 300 kDa | 21.33 | -2.264 to 44.92 | ns | 0.10 |
| PES 1000 kDa vs. HY 100 kDa | 24.86 | 1.266 to 48.45 | * | 0.03 |
| PES 1000 kDa vs. HY 300 kDa | 1.10 | -22.49 to 24.69 | ns | >0.99 |
| RC 100 kDa vs. RC 300 kDa | -6.27 | -29.86 to 17.32 | ns | >0.99 |
| RC 100 kDa vs. HY 100 kDa | -2.74 | -26.33 to 20.85 | ns | >0.99 |
| RC 100 kDa vs. HY 300 kDa | -26.50 | -50.09 to -2.906 | * | 0.02 |
| RC 300 kDa vs. HY 100 kDa | 3.53 | -20.06 to 27.12 | ns | >0.99 |
| RC 300 kDa vs. HY 300 kDa | -20.23 | -43.82 to 3.364 | ns | 0.14 |
| HY 100 kDa vs. HY 300 kDa | -23.76 | -47.35 to -0.165 | * | 0.05 |

**Table S6:** Tukey’s multiple comparison test of DNA removal in retentate samples using different membrane types in a centrifugal ultrafilter. Abbreviations: CI – confidence interval, HY – Hydrosart, PES – polyethersulfone, RC – regenerated cellulose.

| **Membrane types** | **Mean Diff.** | **95% CI of diff.** | **Summary** | **Adjusted p Value** |
| --- | --- | --- | --- | --- |
| PES 100 kDa (+R) vs. PES 100 kDa | 5.62 | -19.76 to 31.00 | ns | 0.9975 |
| PES 100 kDa (+R) vs. PES 300 kDa (+R) | -8.88 | -34.26 to 16.50 | ns | 0.9547 |
| PES 100 kDa (+R) vs. PES 300 kDa | 13.04 | -12.34 to 38.42 | ns | 0.7247 |
| PES 100 kDa (+R) vs. PES 1000 kDa | 3.31 | -22.07 to 28.69 | ns | >0.9999 |
| PES 100 kDa (+R) vs. RC 100 kDa | -11.89 | -37.27 to 13.49 | ns | 0.8089 |
| PES 100 kDa (+R) vs. RC 300 kDa | 1.91 | -23.47 to 27.29 | ns | >0.9999 |
| PES 100 kDa (+R) vs. HY 100 kDa | 23.94 | -1.441 to 49.32 | ns | 0.0759 |
| PES 100 kDa (+R) vs. HY 300 kDa | -7.60 | -32.98 to 17.78 | ns | 0.9818 |
| PES 100 kDa vs. PES 300 kDa (+R) | -14.50 | -39.88 to 10.88 | ns | 0.6054 |
| PES 100 kDa vs. PES 300 kDa | 7.42 | -17.96 to 32.80 | ns | 0.9843 |
| PES 100 kDa vs. PES 1000 kDa | -2.31 | -27.69 to 23.07 | ns | >0.9999 |
| PES 100 kDa vs. RC 100 kDa | -17.51 | -42.89 to 7.871 | ns | 0.3648 |
| PES 100 kDa vs. RC 300 kDa | -3.71 | -29.09 to 21.67 | ns | 0.9999 |
| PES 100 kDa vs. HY 100 kDa | 18.32 | -7.061 to 43.70 | ns | 0.3093 |
| PES 100 kDa vs. HY 300 kDa | -13.22 | -38.60 to 12.16 | ns | 0.7105 |
| PES 300 kDa (+R) vs. PES 300 kDa | 21.92 | -3.461 to 47.30 | ns | 0.1317 |
| PES 300 kDa (+R) vs. PES 1000 kDa | 12.19 | -13.19 to 37.57 | ns | 0.7881 |
| PES 300 kDa (+R) vs. RC 100 kDa | -3.01 | -28.39 to 22.37 | ns | >0.9999 |
| PES 300 kDa (+R) vs. RC 300 kDa | 10.79 | -14.59 to 36.17 | ns | 0.8761 |
| PES 300 kDa (+R) vs. HY 100 kDa | 32.82 | 7.439 to 58.20 | ** | 0.0047 |
| PES 300 kDa (+R) vs. HY 300 kDa | 1.28 | -24.10 to 26.66 | ns | >0.9999 |
| PES 300 kDa vs. PES 1000 kDa | -9.73 | -35.11 to 15.65 | ns | 0.9258 |
| PES 300 kDa vs. RC 100 kDa | -24.93 | -50.31 to 0.4513 | ns | 0.0571 |
| PES 300 kDa vs. RC 300 kDa | -11.13 | -36.51 to 14.25 | ns | 0.8570 |
| PES 300 kDa vs. HY 100 kDa | 10.90 | -14.48 to 36.28 | ns | 0.8701 |
| PES 300 kDa vs. HY 300 kDa | -20.64 | -46.02 to 4.741 | ns | 0.1821 |
| PES 1000 kDa vs. RC 100 kDa | -15.20 | -40.58 to 10.18 | ns | 0.5467 |
| PES 1000 kDa vs. RC 300 kDa | -1.40 | -26.78 to 23.98 | ns | >0.9999 |
| PES 1000 kDa vs. HY 100 kDa | 20.63 | -4.751 to 46.01 | ns | 0.1826 |
| PES 1000 kDa vs. HY 300 kDa | -10.91 | -36.29 to 14.47 | ns | 0.8695 |
| RC 100 kDa vs. RC 300 kDa | 13.80 | -11.58 to 39.18 | ns | 0.6636 |
| RC 100 kDa vs. HY 100 kDa | 35.83 | 10.45 to 61.21 | ** | 0.0017 |
| RC 100 kDa vs. HY 300 kDa | 4.29 | -21.09 to 29.67 | ns | 0.9996 |
| RC 300 kDa vs. HY 100 kDa | 22.03 | -3.351 to 47.41 | ns | 0.1279 |
| RC 300 kDa vs. HY 300 kDa | -9.51 | -34.89 to 15.87 | ns | 0.9342 |
| HY 100 kDa vs. HY 300 kDa | -31.54 | -56.92 to -6.159 | ** | 0.0071 |

**Table S7:** Tukey’s multiple comparison test of infectious LV recovery in retentate samples using different membrane types in a stirred cell. Abbreviations: CI – confidence interval, HY – Hydrosart, PES – polyethersulfone, RC – regenerated cellulose.

| **Membrane types** | **Mean Diff.** | **95% CI of diff.** | **Summary** | **Adjusted p Value** |
| --- | --- | --- | --- | --- |
| PES 100 kDa (+R) vs. PES 100 kDa | 43.70 | 21.36 to 66.04 | *** | <0.001 |
| PES 100 kDa (+R) vs. PES 300 kDa (+R) | 18.04 | -4.296 to 40.38 | ns | 0.19 |
| PES 100 kDa (+R) vs. PES 300 kDa | 33.19 | 10.85 to 55.53 | *** | <0.001 |
| PES 100 kDa (+R) vs. PES 1000 kDa | 66.25 | 43.91 to 88.59 | *** | <0.001 |
| PES 100 kDa (+R) vs. RC 100 kDa | -8.000 | -30.34 to 14.34 | ns | 0.95 |
| PES 100 kDa (+R) vs. RC 300 kDa | -2.090 | -24.43 to 20.25 | ns | >0.99 |
| PES 100 kDa (+R) vs. HY 100 kDa | 9.850 | -12.49 to 32.19 | ns | 0.85 |
| PES 100 kDa (+R) vs. HY 300 kDa | -6.650 | -28.99 to 15.69 | ns | 0.98 |
| PES 100 kDa vs. PES 300 kDa (+R) | -25.66 | -48.00 to -3.324 | * | 0.02 |
| PES 100 kDa vs. PES 300 kDa | -10.51 | -32.85 to 11.83 | ns | 0.81 |
| PES 100 kDa vs. PES 1000 kDa | 22.55 | 0.2139 to 44.89 | * | 0.05 |
| PES 100 kDa vs. RC 100 kDa | -51.70 | -74.04 to -29.36 | *** | <0.001 |
| PES 100 kDa vs. RC 300 kDa | -45.79 | -68.13 to -23.45 | *** | <0.001 |
| PES 100 kDa vs. HY 100 kDa | -33.85 | -56.19 to -11.51 | *** | <0.001 |
| PES 100 kDa vs. HY 300 kDa | -50.35 | -72.69 to -28.01 | *** | <0.001 |
| PES 300 kDa (+R) vs. PES 300 kDa | 15.15 | -7.186 to 37.49 | ns | 0.39 |
| PES 300 kDa (+R) vs. PES 1000 kDa | 48.21 | 25.87 to 70.55 | *** | <0.001 |
| PES 300 kDa (+R) vs. RC 100 kDa | -26.04 | -48.38 to -3.704 | * | 0.01 |
| PES 300 kDa (+R) vs. RC 300 kDa | -20.13 | -42.47 to 2.206 | ns | 0.10 |
| PES 300 kDa (+R) vs. HY 100 kDa | -8.190 | -30.53 to 14.15 | ns | 0.94 |
| PES 300 kDa (+R) vs. HY 300 kDa | -24.69 | -47.03 to -2.354 | * | 0.02 |
| PES 300 kDa vs. PES 1000 kDa | 33.06 | 10.72 to 55.40 | *** | <0.001 |
| PES 300 kDa vs. RC 100 kDa | -41.19 | -63.53 to -18.85 | *** | <0.001 |
| PES 300 kDa vs. RC 300 kDa | -35.28 | -57.62 to -12.94 | *** | <0.001 |
| PES 300 kDa vs. HY 100 kDa | -23.34 | -45.68 to -1.004 | * | 0.04 |
| PES 300 kDa vs. HY 300 kDa | -39.84 | -62.18 to -17.50 | *** | <0.001 |
| PES 1000 kDa vs. RC 100 kDa | -74.25 | -96.59 to -51.91 | *** | <0.001 |
| PES 1000 kDa vs. RC 300 kDa | -68.34 | -90.68 to -46.00 | *** | <0.001 |
| PES 1000 kDa vs. HY 100 kDa | -56.40 | -78.74 to -34.06 | *** | <0.001 |
| PES 1000 kDa vs. HY 300 kDa | -72.90 | -95.24 to -50.56 | *** | <0.001 |
| RC 100 kDa vs. RC 300 kDa | 5.910 | -16.43 to 28.25 | ns | >0.99 |
| RC 100 kDa vs. HY 100 kDa | 17.85 | -4.486 to 40.19 | ns | 0.20 |
| RC 100 kDa vs. HY 300 kDa | 1.350 | -20.99 to 23.69 | ns | >0.99 |
| RC 300 kDa vs. HY 100 kDa | 11.94 | -10.40 to 34.28 | ns | 0.68 |
| RC 300 kDa vs. HY 300 kDa | -4.560 | -26.90 to 17.78 | ns | >0.99 |
| HY 100 kDa vs. HY 300 kDa | -16.50 | -38.84 to 5.836 | ns | 0.28 |

**Table S8:** Tukey’s multiple comparison test of protein removal in retentate samples using different membrane types in a stirred cell. Abbreviations: CI – confidence interval, HY – Hydrosart, PES – polyethersulfone, RC – regenerated cellulose.

| **Membrane types** | **Mean Diff.** | **95% CI of diff.** | **Summary** | **Adjusted p Value** |
| --- | --- | --- | --- | --- |
| PES 100 kDa (+R) vs. PES 100 kDa | -44.59 | -61.91 to -27.27 | *** | <0.001 |
| PES 100 kDa (+R) vs. PES 300 kDa (+R) | -23.31 | -40.63 to -5.990 | ** | 0.003 |
| PES 100 kDa (+R) vs. PES 300 kDa | -17.73 | -35.05 to -0.410 | * | 0.04 |
| PES 100 kDa (+R) vs. PES 1000 kDa | -64.16 | -81.48 to -46.84 | *** | <0.001 |
| PES 100 kDa (+R) vs. RC 100 kDa | 8.65 | -8.670 to 25.97 | ns | 0.75 |
| PES 100 kDa (+R) vs. RC 300 kDa | 0.90 | -16.42 to 18.22 | ns | >0.99 |
| PES 100 kDa (+R) vs. HY 100 kDa | -5.52 | -22.84 to 11.80 | ns | 0.97 |
| PES 100 kDa (+R) vs. HY 300 kDa | -0.55 | -17.87 to 16.77 | ns | >0.99 |
| PES 100 kDa vs. PES 300 kDa (+R) | 21.28 | 3.960 to 38.60 | ** | 0.008 |
| PES 100 kDa vs. PES 300 kDa | 26.86 | 9.540 to 44.18 | *** | <0.001 |
| PES 100 kDa vs. PES 1000 kDa | -19.57 | -36.89 to -2.250 | * | 0.02 |
| PES 100 kDa vs. RC 100 kDa | 53.24 | 35.92 to 70.56 | *** | <0.001 |
| PES 100 kDa vs. RC 300 kDa | 45.49 | 28.17 to 62.81 | *** | <0.001 |
| PES 100 kDa vs. HY 100 kDa | 39.07 | 21.75 to 56.39 | *** | <0.001 |
| PES 100 kDa vs. HY 300 kDa | 44.04 | 26.72 to 61.36 | *** | <0.001 |
| PES 300 kDa (+R) vs. PES 300 kDa | 5.58 | -11.74 to 22.90 | ns | 0.97 |
| PES 300 kDa (+R) vs. PES 1000 kDa | -40.85 | -58.17 to -23.53 | *** | <0.001 |
| PES 300 kDa (+R) vs. RC 100 kDa | 31.96 | 14.64 to 49.28 | *** | <0.001 |
| PES 300 kDa (+R) vs. RC 300 kDa | 24.21 | 6.890 to 41.53 | ** | 0.002 |
| PES 300 kDa (+R) vs. HY 100 kDa | 17.79 | 0.4702 to 35.11 | * | 0.04 |
| PES 300 kDa (+R) vs. HY 300 kDa | 22.76 | 5.440 to 40.08 | ** | 0.004 |
| PES 300 kDa vs. PES 1000 kDa | -46.43 | -63.75 to -29.11 | *** | <0.001 |
| PES 300 kDa vs. RC 100 kDa | 26.38 | 9.060 to 43.70 | *** | <0.001 |
| PES 300 kDa vs. RC 300 kDa | 18.63 | 1.310 to 35.95 | * | 0.03 |
| PES 300 kDa vs. HY 100 kDa | 12.21 | -5.110 to 29.53 | ns | 0.34 |
| PES 300 kDa vs. HY 300 kDa | 17.18 | -0.1398 to 34.50 | ns | 0.05 |
| PES 1000 kDa vs. RC 100 kDa | 72.81 | 55.49 to 90.13 | *** | <0.001 |
| PES 1000 kDa vs. RC 300 kDa | 65.06 | 47.74 to 82.38 | *** | <0.001 |
| PES 1000 kDa vs. HY 100 kDa | 58.64 | 41.32 to 75.96 | *** | <0.001 |
| PES 1000 kDa vs. HY 300 kDa | 63.61 | 46.29 to 80.93 | *** | <0.001 |
| RC 100 kDa vs. RC 300 kDa | -7.75 | -25.07 to 9.570 | ns | 0.84 |
| RC 100 kDa vs. HY 100 kDa | -14.17 | -31.49 to 3.150 | ns | 0.18 |
| RC 100 kDa vs. HY 300 kDa | -9.20 | -26.52 to 8.120 | ns | 0.69 |
| RC 300 kDa vs. HY 100 kDa | -6.42 | -23.74 to 10.90 | ns | 0.94 |
| RC 300 kDa vs. HY 300 kDa | -1.45 | -18.77 to 15.87 | ns | >0.99 |
| HY 100 kDa vs. HY 300 kDa | 4.97 | -12.35 to 22.29 | ns | 0.99 |

**Table S9:** Tukey’s multiple comparison test of DNA removal in retentate samples using different membrane types in a stirred cell. Abbreviations: CI – confidence interval, HY – Hydrosart, PES – polyethersulfone, RC – regenerated cellulose.

| **Membrane types** | **Mean Diff.** | **95% CI of diff.** | **Summary** | **Adjusted p Value** |
| --- | --- | --- | --- | --- |
| PES 100 kDa (+R) vs. PES 100 kDa | -28.23 | -48.00 to -8.460 | ** | 0.001 |
| PES 100 kDa (+R) vs. PES 300 kDa (+R) | -10.64 | -30.41 to 9.130 | ns | 0.68 |
| PES 100 kDa (+R) vs. PES 300 kDa | -11.60 | -31.37 to 8.170 | ns | 0.57 |
| PES 100 kDa (+R) vs. PES 1000 kDa | -42.53 | -62.30 to -22.76 | *** | <0.001 |
| PES 100 kDa (+R) vs. RC 100 kDa | 16.19 | -3.580 to 35.96 | ns | 0.18 |
| PES 100 kDa (+R) vs. RC 300 kDa | 5.84 | -13.93 to 25.61 | ns | 0.98 |
| PES 100 kDa (+R) vs. HY 100 kDa | 2.00 | -17.77 to 21.77 | ns | >0.99 |
| PES 100 kDa (+R) vs. HY 300 kDa | 1.080 | -18.69 to 20.85 | ns | >0.99 |
| PES 100 kDa vs. PES 300 kDa (+R) | 17.59 | -2.180 to 37.36 | ns | 0.11 |
| PES 100 kDa vs. PES 300 kDa | 16.63 | -3.140 to 36.40 | ns | 0.15 |
| PES 100 kDa vs. PES 1000 kDa | -14.30 | -34.07 to 5.470 | ns | 0.31 |
| PES 100 kDa vs. RC 100 kDa | 44.42 | 24.65 to 64.19 | *** | <0.001 |
| PES 100 kDa vs. RC 300 kDa | 34.07 | 14.30 to 53.84 | *** | <0.001 |
| PES 100 kDa vs. HY 100 kDa | 30.23 | 10.46 to 50.00 | *** | <0.001 |
| PES 100 kDa vs. HY 300 kDa | 29.31 | 9.540 to 49.08 | *** | <0.001 |
| PES 300 kDa (+R) vs. PES 300 kDa | -0.96 | -20.73 to 18.81 | ns | >0.99 |
| PES 300 kDa (+R) vs. PES 1000 kDa | -31.89 | -51.66 to -12.12 | *** | <0.001 |
| PES 300 kDa (+R) vs. RC 100 kDa | 26.83 | 7.060 to 46.60 | ** | 0.003 |
| PES 300 kDa (+R) vs. RC 300 kDa | 16.48 | -3.290 to 36.25 | ns | 0.16 |
| PES 300 kDa (+R) vs. HY 100 kDa | 12.64 | -7.130 to 32.41 | ns | 0.46 |
| PES 300 kDa (+R) vs. HY 300 kDa | 11.72 | -8.050 to 31.49 | ns | 0.56 |
| PES 300 kDa vs. PES 1000 kDa | -30.93 | -50.70 to -11.16 | *** | <0.001 |
| PES 300 kDa vs. RC 100 kDa | 27.79 | 8.020 to 47.56 | ** | 0.002 |
| PES 300 kDa vs. RC 300 kDa | 17.44 | -2.330 to 37.21 | ns | 0.12 |
| PES 300 kDa vs. HY 100 kDa | 13.60 | -6.170 to 33.37 | ns | 0.37 |
| PES 300 kDa vs. HY 300 kDa | 12.68 | -7.090 to 32.45 | ns | 0.46 |
| PES 1000 kDa vs. RC 100 kDa | 58.72 | 38.95 to 78.49 | *** | <0.001 |
| PES 1000 kDa vs. RC 300 kDa | 48.37 | 28.60 to 68.14 | *** | <0.001 |
| PES 1000 kDa vs. HY 100 kDa | 44.53 | 24.76 to 64.30 | *** | <0.001 |
| PES 1000 kDa vs. HY 300 kDa | 43.61 | 23.84 to 63.38 | *** | <0.001 |
| RC 100 kDa vs. RC 300 kDa | -10.35 | -30.12 to 9.420 | ns | 0.71 |
| RC 100 kDa vs. HY 100 kDa | -14.19 | -33.96 to 5.580 | ns | 0.32 |
| RC 100 kDa vs. HY 300 kDa | -15.11 | -34.88 to 4.660 | ns | 0.24 |
| RC 300 kDa vs. HY 100 kDa | -3.84 | -23.61 to 15.93 | ns | >0.99 |
| RC 300 kDa vs. HY 300 kDa | -4.76 | -24.53 to 15.01 | ns | >0.99 |
| HY 100 kDa vs. HY 300 kDa | -0.90 | -20.69 to 18.85 | ns | >0.99 |

**Table S10:** Tukey’s multiple comparison test of LV particle recovery in retentate samples using different membrane types in a crossflow cassette. Abbreviations: CI – confidence interval, HY – Hydrosart, PES – polyethersulfone, RC – regenerated cellulose.

| **Membrane types** | **Mean Diff.** | **95% CI of diff.** | **Summary** | **Adjusted p Value** |
| --- | --- | --- | --- | --- |
| PES 100 kDa (+R) vs. PES 100 kDa | 9 | -13.74 to 27.16 | ns | 0.97 |
| PES 100 kDa (+R) vs. PES 300 kDa (+R) | -17.01 | -37.46 to 3.443 | ns | 0.16 |
| PES 100 kDa (+R) vs. PES 300 kDa | -5.96 | -26.41 to 14.49 | ns | 0.98 |
| PES 100 kDa (+R) vs. PES 1000 kDa | 18.93 | -1.523 to 39.38 | ns | 0.09 |
| PES 100 kDa (+R) vs. RC 100 kDa | -13.97 | -34.42 to 6.483 | ns | 0.38 |
| PES 100 kDa (+R) vs. RC 300 kDa | -4.81 | -25.26 to 15.64 | ns | >0.99 |
| PES 100 kDa (+R) vs. HY 100 kDa | 4.06 | -16.39 to 24.51 | ns | >0.99 |
| PES 100 kDa (+R) vs. HY 300 kDa | -22.62 | -43.07 to -2.167 | * | 0.02 |
| PES 100 kDa vs. PES 300 kDa (+R) | -23.72 | -44.17 to -3.267 | * | 0.01 |
| PES 100 kDa vs. PES 300 kDa | -12.67 | -33.12 to 7.783 | ns | 0.5 |
| PES 100 kDa vs. PES 1000 kDa | 12.22 | -8.233 to 32.67 | ns | 0.55 |
| PES 100 kDa vs. RC 100 kDa | -20.68 | -41.13 to -0.2272 | * | 0.05 |
| PES 100 kDa vs. RC 300 kDa | -11.52 | -31.97 to 8.933 | ns | 0.62 |
| PES 100 kDa vs. HY 100 kDa | -2.65 | -23.10 to 17.80 | ns | >0.99 |
| PES 100 kDa vs. HY 300 kDa | -29.33 | -49.78 to -8.877 | ** | 0.001 |
| PES 300 kDa (+R) vs. PES 300 kDa | 11.05 | -9.403 to 31.50 | ns | 0.67 |
| PES 300 kDa (+R) vs. PES 1000 kDa | 35.94 | 15.49 to 56.39 | *** | <0.001 |
| PES 300 kDa (+R) vs. RC 100 kDa | 3.04 | -17.41 to 23.49 | ns | >0.99 |
| PES 300 kDa (+R) vs. RC 300 kDa | 12.2 | -8.253 to 32.65 | ns | 0.55 |
| PES 300 kDa (+R) vs. HY 100 kDa | 21.07 | 0.6172 to 41.52 | * | 0.04 |
| PES 300 kDa (+R) vs. HY 300 kDa | -5.61 | -26.06 to 14.84 | ns | 0.99 |
| PES 300 kDa vs. PES 1000 kDa | 24.89 | 4.437 to 45.34 | ** | 0.009 |
| PES 300 kDa vs. RC 100 kDa | -8.01 | -28.46 to 12.44 | ns | 0.92 |
| PES 300 kDa vs. RC 300 kDa | 1.15 | -19.30 to 21.60 | ns | >0.99 |
| PES 300 kDa vs. HY 100 kDa | 10.02 | -10.43 to 30.47 | ns | 0.77 |
| PES 300 kDa vs. HY 300 kDa | -16.66 | -37.11 to 3.793 | ns | 0.18 |
| PES 1000 kDa vs. RC 100 kDa | -32.9 | -53.35 to -12.45 | *** | <0.001 |
| PES 1000 kDa vs. RC 300 kDa | -23.74 | -44.19 to -3.287 | * | 0.01 |
| PES 1000 kDa vs. HY 100 kDa | -14.87 | -35.32 to 5.583 | ns | 0.3 |
| PES 1000 kDa vs. HY 300 kDa | -41.55 | -62.00 to -21.10 | *** | <0.001 |
| RC 100 kDa vs. RC 300 kDa | 9.16 | -11.29 to 29.61 | ns | 0.84 |
| RC 100 kDa vs. HY 100 kDa | 18.03 | -2.423 to 38.48 | ns | 0.12 |
| RC 100 kDa vs. HY 300 kDa | -8.65 | -29.10 to 11.80 | ns | 0.88 |
| RC 300 kDa vs. HY 100 kDa | 8.87 | -11.58 to 29.32 | ns | 0.86 |
| RC 300 kDa vs. HY 300 kDa | -17.81 | -38.26 to 2.643 | ns | 0.13 |
| HY 100 kDa vs. HY 300 kDa | -26.68 | -47.13 to -6.227 | ** | 0.004 |

**Table S11:** Tukey’s multiple comparison test of infectious LV recovery in retentate samples using different membrane types in a crossflow cassette. Abbreviations: CI – confidence interval. HY – Hydrosart. PES – polyethersulfone. RC – regenerated cellulose.

| **Membrane types** | **Mean Diff.** | **95% CI of diff.** | **Summary** | **Adjusted p Value** |
| --- | --- | --- | --- | --- |
| PES 100 kDa (+R) vs. PES 100 kDa | 6.18 | 0.4587 to 11.90 | * | 0.03 |
| PES 100 kDa (+R) vs. PES 300 kDa (+R) | 13.66 | 7.939 to 19.38 | *** | <0.001 |
| PES 100 kDa (+R) vs. PES 300 kDa | 15.17 | 9.449 to 20.89 | *** | <0.001 |
| PES 100 kDa (+R) vs. PES 1000 kDa | 23.63 | 17.91 to 29.35 | *** | <0.001 |
| PES 100 kDa (+R) vs. RC 100 kDa | 7.19 | 1.469 to 12.91 | ** | 0.006 |
| PES 100 kDa (+R) vs. RC 300 kDa | 9.16 | 3.439 to 14.88 | *** | <0.001 |
| PES 100 kDa (+R) vs. HY 100 kDa | 13.2 | 7.479 to 18.92 | *** | <0.001 |
| PES 100 kDa (+R) vs. HY 300 kDa | -7.16 | -12.88 to -1.439 | ** | 0.007 |
| PES 100 kDa vs. PES 300 kDa (+R) | 7.48 | 1.759 to 13.20 | ** | 0.004 |
| PES 100 kDa vs. PES 300 kDa | 8.99 | 3.269 to 14.71 | *** | <0.001 |
| PES 100 kDa vs. PES 1000 kDa | 17.45 | 11.73 to 23.17 | *** | <0.001 |
| PES 100 kDa vs. RC 100 kDa | 1.01 | -4.711 to 6.731 | ns | >0.99 |
| PES 100 kDa vs. RC 300 kDa | 2.98 | -2.741 to 8.701 | ns | 0.71 |
| PES 100 kDa vs. HY 100 kDa | 7.02 | 1.299 to 12.74 | ** | 0.008 |
| PES 100 kDa vs. HY 300 kDa | -13.34 | -19.06 to -7.619 | *** | <0.001 |
| PES 300 kDa (+R) vs. PES 300 kDa | 1.51 | -4.211 to 7.231 | ns | >0.99 |
| PES 300 kDa (+R) vs. PES 1000 kDa | 9.97 | 4.249 to 15.69 | *** | <0.001 |
| PES 300 kDa (+R) vs. RC 100 kDa | -6.47 | -12.19 to -0.7487 | * | 0.02 |
| PES 300 kDa (+R) vs. RC 300 kDa | -4.5 | -10.22 to 1.221 | ns | 0.21 |
| PES 300 kDa (+R) vs. HY 100 kDa | -0.46 | -6.181 to 5.261 | ns | >0.99 |
| PES 300 kDa (+R) vs. HY 300 kDa | -20.82 | -26.54 to -15.10 | *** | <0.001 |
| PES 300 kDa vs. PES 1000 kDa | 8.46 | 2.739 to 14.18 | *** | <0.001 |
| PES 300 kDa vs. RC 100 kDa | -7.98 | -13.70 to -2.259 | ** | 0.002 |
| PES 300 kDa vs. RC 300 kDa | -6.01 | -11.73 to -0.2887 | * | 0.03 |
| PES 300 kDa vs. HY 100 kDa | -1.97 | -7.691 to 3.751 | ns | 0.96 |
| PES 300 kDa vs. HY 300 kDa | -22.33 | -28.05 to -16.61 | *** | <0.001 |
| PES 1000 kDa vs. RC 100 kDa | -16.44 | -22.16 to -10.72 | *** | <0.001 |
| PES 1000 kDa vs. RC 300 kDa | -14.47 | -20.19 to -8.749 | *** | <0.001 |
| PES 1000 kDa vs. HY 100 kDa | -10.43 | -16.15 to -4.709 | *** | <0.001 |
| PES 1000 kDa vs. HY 300 kDa | -30.79 | -36.51 to -25.07 | *** | <0.001 |
| RC 100 kDa vs. RC 300 kDa | 1.97 | -3.751 to 7.691 | ns | 0.96 |
| RC 100 kDa vs. HY 100 kDa | 6.01 | 0.2887 to 11.73 | * | 0.03 |
| RC 100 kDa vs. HY 300 kDa | -14.35 | -20.07 to -8.629 | *** | <0.001 |
| RC 300 kDa vs. HY 100 kDa | 4.04 | -1.681 to 9.761 | ns | 0.34 |
| RC 300 kDa vs. HY 300 kDa | -16.32 | -22.04 to -10.60 | *** | <0.001 |
| HY 100 kDa vs. HY 300 kDa | -20.36 | -26.08 to -14.64 | *** | <0.001 |

**Table S12:** Tukey’s multiple comparison test of protein removal in retentate samples using different membrane types in a crossflow cassette. Abbreviations: CI – confidence interval. HY – Hydrosart. PES – polyethersulfone. RC – regenerated cellulose.

| **Membrane types** | **Mean Diff.** | **95% CI of diff.** | **Summary** | **Adjusted p Value** |
| --- | --- | --- | --- | --- |
| PES 100 kDa (+R) vs. PES 100 kDa | 14.96 | 4.847 to 25.07 | *** | <0.001 |
| PES 100 kDa (+R) vs. PES 300 kDa (+R) | 26.09 | 15.98 to 36.20 | *** | <0.001 |
| PES 100 kDa (+R) vs. PES 300 kDa | 6.11 | -4.003 to 16.22 | ns | 0.54 |
| PES 100 kDa (+R) vs. PES 1000 kDa | -49.17 | -59.28 to -39.06 | *** | <0.001 |
| PES 100 kDa (+R) vs. RC 100 kDa | 18.85 | 8.737 to 28.96 | *** | <0.001 |
| PES 100 kDa (+R) vs. RC 300 kDa | 26.89 | 16.78 to 37.00 | *** | <0.001 |
| PES 100 kDa (+R) vs. HY 100 kDa | -6.71 | -16.82 to 3.403 | ns | 0.41 |
| PES 100 kDa (+R) vs. HY 300 kDa | -5.06 | -15.17 to 5.053 | ns | 0.75 |
| PES 100 kDa vs. PES 300 kDa (+R) | 11.13 | 1.017 to 21.24 | * | 0.02 |
| PES 100 kDa vs. PES 300 kDa | -8.85 | -18.96 to 1.263 | ns | 0.12 |
| PES 100 kDa vs. PES 1000 kDa | -64.13 | -74.24 to -54.02 | *** | <0.001 |
| PES 100 kDa vs. RC 100 kDa | 3.89 | -6.223 to 14.00 | ns | 0.92 |
| PES 100 kDa vs. RC 300 kDa | 11.93 | 1.817 to 22.04 | * | 0.01 |
| PES 100 kDa vs. HY 100 kDa | -21.67 | -31.78 to -11.56 | *** | <0.001 |
| PES 100 kDa vs. HY 300 kDa | -20.02 | -30.13 to -9.907 | *** | <0.001 |
| PES 300 kDa (+R) vs. PES 300 kDa | -19.98 | -30.09 to -9.867 | *** | <0.001 |
| PES 300 kDa (+R) vs. PES 1000 kDa | -75.26 | -85.37 to -65.15 | *** | <0.001 |
| PES 300 kDa (+R) vs. RC 100 kDa | -7.24 | -17.35 to 2.873 | ns | 0.32 |
| PES 300 kDa (+R) vs. RC 300 kDa | 0.80 | -9.313 to 10.91 | ns | >0.99 |
| PES 300 kDa (+R) vs. HY 100 kDa | -32.80 | -42.91 to -22.69 | *** | <0.001 |
| PES 300 kDa (+R) vs. HY 300 kDa | -31.15 | -41.26 to -21.04 | *** | <0.001 |
| PES 300 kDa vs. PES 1000 kDa | -55.28 | -65.39 to -45.17 | *** | <0.001 |
| PES 300 kDa vs. RC 100 kDa | 12.74 | 2.627 to 22.85 | ** | 0.006 |
| PES 300 kDa vs. RC 300 kDa | 20.78 | 10.67 to 30.89 | *** | <0.001 |
| PES 300 kDa vs. HY 100 kDa | -12.82 | -22.93 to -2.707 | ** | 0.006 |
| PES 300 kDa vs. HY 300 kDa | -11.17 | -21.28 to -1.057 | * | 0.02 |
| PES 1000 kDa vs. RC 100 kDa | 68.02 | 57.91 to 78.13 | *** | <0.001 |
| PES 1000 kDa vs. RC 300 kDa | 76.06 | 65.95 to 86.17 | *** | <0.001 |
| PES 1000 kDa vs. HY 100 kDa | 42.46 | 32.35 to 52.57 | *** | <0.001 |
| PES 1000 kDa vs. HY 300 kDa | 44.11 | 34.00 to 54.22 | *** | <0.001 |
| RC 100 kDa vs. RC 300 kDa | 8.04 | -2.073 to 18.15 | ns | 0.20 |
| RC 100 kDa vs. HY 100 kDa | -25.56 | -35.67 to -15.45 | *** | <0.001 |
| RC 100 kDa vs. HY 300 kDa | -23.91 | -34.02 to -13.80 | *** | <0.001 |
| RC 300 kDa vs. HY 100 kDa | -33.60 | -43.71 to -23.49 | *** | <0.001 |
| RC 300 kDa vs. HY 300 kDa | -31.95 | -42.06 to -21.84 | *** | <0.001 |
| HY 100 kDa vs. HY 300 kDa | 1.65 | -8.463 to 11.76 | ns | >0.99 |

**Table S13:** Tukey’s multiple comparison test of DNA removal in retentate samples using different membrane types in a crossflow cassette. Abbreviations: CI – confidence interval. HY – Hydrosart. PES – polyethersulfone. RC – regenerated cellulose.

| **Membrane types** | **Mean Diff.** | **95% CI of diff.** | **Summary** | **Adjusted p Value** |
| --- | --- | --- | --- | --- |
| PES 100 kDa (+R) vs. PES 100 kDa | -4.25 | -25.27 to 16.77 | ns | >0.99 |
| PES 100 kDa (+R) vs. PES 300 kDa (+R) | 27.38 | 6.357 to 48.40 | ** | 0.004 |
| PES 100 kDa (+R) vs. PES 300 kDa | -4.650 | -25.67 to 16.37 | ns | >0.99 |
| PES 100 kDa (+R) vs. PES 1000 kDa | -17.46 | -38.48 to 3.563 | ns | 0.16 |
| PES 100 kDa (+R) vs. RC 100 kDa | 32.16 | 11.14 to 53.18 | *** | <0.001 |
| PES 100 kDa (+R) vs. RC 300 kDa | 29.32 | 8.297 to 50.34 | ** | 0.002 |
| PES 100 kDa (+R) vs. HY 100 kDa | 9.13 | -11.89 to 30.15 | ns | 0.86 |
| PES 100 kDa (+R) vs. HY 300 kDa | -4.50 | -25.52 to 16.52 | ns | >0.99 |
| PES 100 kDa vs. PES 300 kDa (+R) | 31.63 | 10.61 to 52.65 | *** | <0.001 |
| PES 100 kDa vs. PES 300 kDa | -0.40 | -21.42 to 20.62 | ns | >0.99 |
| PES 100 kDa vs. PES 1000 kDa | -13.21 | -34.23 to 7.813 | ns | 0.48 |
| PES 100 kDa vs. RC 100 kDa | 36.41 | 15.39 to 57.43 | *** | <0.001 |
| PES 100 kDa vs. RC 300 kDa | 33.57 | 12.55 to 54.59 | *** | <0.001 |
| PES 100 kDa vs. HY 100 kDa | 13.38 | -7.643 to 34.40 | ns | 0.47 |
| PES 100 kDa vs. HY 300 kDa | -0.25 | -21.27 to 20.77 | ns | >0.99 |
| PES 300 kDa (+R) vs. PES 300 kDa | -32.03 | -53.05 to -11.01 | *** | <0.001 |
| PES 300 kDa (+R) vs. PES 1000 kDa | -44.84 | -65.86 to -23.82 | *** | <0.001 |
| PES 300 kDa (+R) vs. RC 100 kDa | 4.78 | -16.24 to 25.80 | ns | >0.99 |
| PES 300 kDa (+R) vs. RC 300 kDa | 1.94 | -19.08 to 22.96 | ns | >0.99 |
| PES 300 kDa (+R) vs. HY 100 kDa | -18.25 | -39.27 to 2.773 | ns | 0.13 |
| PES 300 kDa (+R) vs. HY 300 kDa | -31.88 | -52.90 to -10.86 | *** | <0.001 |
| PES 300 kDa vs. PES 1000 kDa | -12.81 | -33.83 to 8.213 | ns | 0.52 |
| PES 300 kDa vs. RC 100 kDa | 36.81 | 15.79 to 57.83 | *** | <0.001 |
| PES 300 kDa vs. RC 300 kDa | 33.97 | 12.95 to 54.99 | *** | <0.001 |
| PES 300 kDa vs. HY 100 kDa | 13.78 | -7.243 to 34.80 | ns | 0.43 |
| PES 300 kDa vs. HY 300 kDa | 0.15 | -20.87 to 21.17 | ns | >0.99 |
| PES 1000 kDa vs. RC 100 kDa | 49.62 | 28.60 to 70.64 | *** | <0.001 |
| PES 1000 kDa vs. RC 300 kDa | 46.78 | 25.76 to 67.80 | *** | <0.001 |
| PES 1000 kDa vs. HY 100 kDa | 26.59 | 5.567 to 47.61 | ** | 0.006 |
| PES 1000 kDa vs. HY 300 kDa | 12.96 | -8.063 to 33.98 | ns | 0.51 |
| RC 100 kDa vs. RC 300 kDa | -2.84 | -23.86 to 18.18 | ns | >0.99 |
| RC 100 kDa vs. HY 100 kDa | -23.03 | -44.05 to -2.007 | * | 0.02 |
| RC 100 kDa vs. HY 300 kDa | -36.66 | -57.68 to -15.64 | *** | <0.001 |
| RC 300 kDa vs. HY 100 kDa | -20.19 | -41.21 to 0.8329 | ns | 0.07 |
| RC 300 kDa vs. HY 300 kDa | -33.82 | -54.84 to -12.80 | *** | <0.001 |
| HY 100 kDa vs. HY 300 kDa | -13.63 | -34.65 to 7.393 | ns | 0.44 |

**Table S14:** Tukey’s multiple comparison test of infectious LV recovery in retentate samples using different membrane types in the three different devices. Abbreviations: CI – confidence interval. HY – Hydrosart. PES – polyethersulfone. RC – regenerated cellulose.

| **Membrane Types** | **Mean Diff.** | **95.00% CI of diff.** | **Summary** | **Adjusted p Value** |
| --- | --- | --- | --- | --- |
| PES 100 kDa (+R) |  |  |  |  |
| Stirred cell vs. Centrifugal ultrafilters | 7.23 | -7.077 to 21.54 | ns | 0.453 |
| Stirred cell vs. Crossflow cassettes | 36.18 | 21.87 to 50.49 | *** | <.001 |
| Centrifugal ultrafilters vs. Crossflow cassettes | 28.95 | 14.64 to 43.26 | *** | <.001 |
|  |  |  |  |  |
| PES 100 kDa |  |  |  |  |
| Stirred cell vs. Centrifugal ultrafilters | -5.58 | -19.89 to 8.727 | ns | 0.622 |
| Stirred cell vs. Crossflow cassettes | -1.34 | -15.65 to 12.97 | ns | 0.973 |
| Centrifugal ultrafilters vs. Crossflow cassettes | 4.24 | -10.07 to 18.55 | ns | 0.76 |
|  |  |  |  |  |
|  |  |  |  |  |
| PES 300 kDa (+R) |  |  |  |  |
| Stirred cell vs. Centrifugal ultrafilters | 14.59 | 0.2831 to 28.90 | * | 0.045 |
| Stirred cell vs. Crossflow cassettes | 31.8 | 17.49 to 46.11 | *** | <.001 |
| Centrifugal ultrafilters vs. Crossflow cassettes | 17.21 | 2.903 to 31.52 | * | 0.014 |
|  |  |  |  |  |
| PES 300 kDa |  |  |  |  |
| Stirred cell vs. Centrifugal ultrafilters | 10.55 | -3.757 to 24.86 | ns | 0.189 |
| Stirred cell vs. Crossflow cassettes | 18.16 | 3.853 to 32.47 | ** | 0.009 |
| Centrifugal ultrafilters vs. Crossflow cassettes | 7.61 | -6.697 to 21.92 | ns | 0.416 |
|  |  |  |  |  |
| PES 1000 kDa |  |  |  |  |
| Stirred cell vs. Centrifugal ultrafilters | -11.09 | -25.40 to 3.217 | ns | 0.16 |
| Stirred cell vs. Crossflow cassettes | -6.44 | -20.75 to 7.867 | ns | 0.532 |
| Centrifugal ultrafilters vs. Crossflow cassettes | 4.65 | -9.657 to 18.96 | ns | 0.719 |
|  |  |  |  |  |
| RC 100 kDa |  |  |  |  |
| Stirred cell vs. Centrifugal ultrafilters | 39.03 | 24.72 to 53.34 | *** | <.001 |
| Stirred cell vs. Crossflow cassettes | 51.37 | 37.06 to 65.68 | *** | <.001 |
| Centrifugal ultrafilters vs. Crossflow cassettes | 12.34 | -1.967 to 26.65 | ns | 0.105 |
|  |  |  |  |  |
| RC 300 kDa |  |  |  |  |
| Stirred cell vs. Centrifugal ultrafilters | 34.29 | 19.98 to 48.60 | *** | <.001 |
| Stirred cell vs. Crossflow cassettes | 47.43 | 33.12 to 61.74 | *** | <.001 |
| Centrifugal ultrafilters vs. Crossflow cassettes | 13.14 | -1.167 to 27.45 | ns | 0.078 |
|  |  |  |  |  |
| HY 100 kDa |  |  |  |  |
| Stirred cell vs. Centrifugal ultrafilters | 20.85 | 6.543 to 35.16 | ** | 0.002 |
| Stirred cell vs. Crossflow cassettes | 39.53 | 25.22 to 53.84 | *** | <.001 |
| Centrifugal ultrafilters vs. Crossflow cassettes | 18.68 | 4.373 to 32.99 | ** | 0.007 |
|  |  |  |  |  |
| HY 300 kDa |  |  |  |  |
| Stirred cell vs. Centrifugal ultrafilters | 36.94 | 22.63 to 51.25 | *** | <.001 |
| Stirred cell vs. Crossflow cassettes | 35.67 | 21.36 to 49.98 | *** | <.001 |
| Centrifugal ultrafilters vs. Crossflow cassettes | -1.27 | -15.58 to 13.04 | ns | 0.976 |

**Table S15:** Tukey’s multiple comparison test of protein removal in retentate samples using different membrane types in the three different devices. Abbreviations: CI – confidence interval. HY – Hydrosart. PES – polyethersulfone. RC – regenerated cellulose.

| **Membrane Types** | **Mean Diff.** | **95.00% CI of diff.** | **Summary** | **Adjusted p Value** |
| --- | --- | --- | --- | --- |
| PES 100 kDa (+R) |  |  |  |  |
| Stirred cell vs. Centrifugal ultrafilters | 0.83 | -11.83 to 13.49 | ns | 0.987 |
| Stirred cell vs. Crossflow cassettes | -4.92 | -17.58 to 7.735 | ns | 0.624 |
| Centrifugal ultrafilters vs. Crossflow cassettes | -5.75 | -18.41 to 6.905 | ns | 0.526 |
|  |  |  |  |  |
| PES 100 kDa |  |  |  |  |
| Stirred cell vs. Centrifugal ultrafilters | 33.77 | 21.11 to 46.43 | *** | <0.001 |
| Stirred cell vs. Crossflow cassettes | 54.63 | 41.97 to 67.29 | *** | <0.001 |
| Centrifugal ultrafilters vs. Crossflow cassettes | 20.86 | 8.205 to 33.52 | *** | <0.001 |
|  |  |  |  |  |
| PES 300 kDa (+R) |  |  |  |  |
| Stirred cell vs. Centrifugal ultrafilters | 2.00 | -10.66 to 14.66 | ns | 0.925 |
| Stirred cell vs. Crossflow cassettes | 44.48 | 31.82 to 57.14 | *** | <0.001 |
| Centrifugal ultrafilters vs. Crossflow cassettes | 42.48 | 29.82 to 55.14 | *** | <0.001 |
|  |  |  |  |  |
| PES 300 kDa |  |  |  |  |
| Stirred cell vs. Centrifugal ultrafilters | 3.69 | -8.965 to 16.35 | ns | 0.766 |
| Stirred cell vs. Crossflow cassettes | 18.92 | 6.265 to 31.58 | ** | 0.002 |
| Centrifugal ultrafilters vs. Crossflow cassettes | 15.23 | 2.575 to 27.89 | * | 0.014 |
|  |  |  |  |  |
| PES 1000 kDa |  |  |  |  |
| Stirred cell vs. Centrifugal ultrafilters | 54.39 | 41.73 to 67.05 | *** | <0.001 |
| Stirred cell vs. Crossflow cassettes | 10.07 | -2.585 to 22.73 | ns | 0.145 |
| Centrifugal ultrafilters vs. Crossflow cassettes | -44.32 | -56.98 to -31.66 | *** | <0.001 |
|  |  |  |  |  |
| RC 100 kDa |  |  |  |  |
| Stirred cell vs. Centrifugal ultrafilters | 9.18 | -3.475 to 21.84 | ns | 0.200 |
| Stirred cell vs. Crossflow cassettes | 5.28 | -7.375 to 17.94 | ns | 0.581 |
| Centrifugal ultrafilters vs. Crossflow cassettes | -3.90 | -16.56 to 8.755 | ns | 0.743 |
|  |  |  |  |  |
| RC 300 kDa |  |  |  |  |
| Stirred cell vs. Centrifugal ultrafilters | 10.66 | -1.995 to 23.32 | ns | 0.116 |
| Stirred cell vs. Crossflow cassettes | 21.07 | 8.415 to 33.73 | *** | <0.001 |
| Centrifugal ultrafilters vs. Crossflow cassettes | 10.41 | -2.245 to 23.07 | ns | 0.128 |
|  |  |  |  |  |
| HY 100 kDa |  |  |  |  |
| Stirred cell vs. Centrifugal ultrafilters | 20.61 | 7.955 to 33.27 | *** | <0.001 |
| Stirred cell vs. Crossflow cassettes | -6.11 | -18.77 to 6.545 | ns | 0.485 |
| Centrifugal ultrafilters vs. Crossflow cassettes | -26.72 | -39.38 to -14.06 | *** | <0.001 |
|  |  |  |  |  |
| HY 300 kDa |  |  |  |  |
| Stirred cell vs. Centrifugal ultrafilters | -8.12 | -20.78 to 4.535 | ns | 0.281 |
| Stirred cell vs. Crossflow cassettes | -9.43 | -22.09 to 3.225 | ns | 0.183 |
| Centrifugal ultrafilters vs. Crossflow cassettes | -1.31 | -13.97 to 11.35 | ns | 0.967 |

**Table S16:** Tukey’s multiple comparison test of DNA removal in retentate samples using different membrane types in the three different devices. Abbreviations: CI – confidence interval. HY – Hydrosart. PES – polyethersulfone. RC – regenerated cellulose.

| **Membrane Types** | **Mean Diff.** | **95.00% CI of diff.** | **Summary** | **Adjusted p Value** |
| --- | --- | --- | --- | --- |
| PES 100 kDa (+R) |  |  |  |  |
| Stirred cell vs. Centrifugal ultrafilters | -16.93 | -32.67 to -1.185 | * | 0.032 |
| Stirred cell vs. Crossflow cassettes | -23.15 | -38.89 to -7.405 | ** | 0.002 |
| Centrifugal ultrafilters vs. Crossflow cassettes | -6.22 | -21.96 to 9.525 | ns | 0.615 |
|  |  |  |  |  |
| PES 100 kDa |  |  |  |  |
| Stirred cell vs. Centrifugal ultrafilters | 16.92 | 1.175 to 32.66 | * | 0.032 |
| Stirred cell vs. Crossflow cassettes | 0.83 | -14.91 to 16.57 | ns | 0.991 |
| Centrifugal ultrafilters vs. Crossflow cassettes | -16.09 | -31.83 to -0.3452 | * | 0.044 |
|  |  |  |  |  |
| PES 300 kDa (+R) |  |  |  |  |
| Stirred cell vs. Centrifugal ultrafilters | -15.17 | -30.91 to 0.5748 | ns | 0.061 |
| Stirred cell vs. Crossflow cassettes | 14.87 | -0.8748 to 30.61 | ns | 0.068 |
| Centrifugal ultrafilters vs. Crossflow cassettes | 30.04 | 14.30 to 45.78 | *** | <0.001 |
|  |  |  |  |  |
| PES 300 kDa |  |  |  |  |
| Stirred cell vs. Centrifugal ultrafilters | 7.71 | -8.035 to 23.45 | ns | 0.475 |
| Stirred cell vs. Crossflow cassettes | -16.20 | -31.94 to -0.455 | * | 0.042 |
| Centrifugal ultrafilters vs. Crossflow cassettes | -23.91 | -39.65 to -8.165 | ** | 0.001 |
|  |  |  |  |  |
| PES 1000 kDa |  |  |  |  |
| Stirred cell vs. Centrifugal ultrafilters | 28.91 | 13.17 to 44.65 | *** | <0.001 |
| Stirred cell vs. Crossflow cassettes | 1.92 | -13.82 to 17.66 | ns | 0.954 |
| Centrifugal ultrafilters vs. Crossflow cassettes | -26.99 | -42.73 to -11.25 | *** | <0.001 |
|  |  |  |  |  |
| RC 100 kDa |  |  |  |  |
| Stirred cell vs. Centrifugal ultrafilters | -45.01 | -60.75 to -29.27 | *** | <0.001 |
| Stirred cell vs. Crossflow cassettes | -7.18 | -22.92 to 8.565 | ns | 0.524 |
| Centrifugal ultrafilters vs. Crossflow cassettes | 37.83 | 22.09 to 53.57 | *** | <0.001 |
|  |  |  |  |  |
| RC 300 kDa |  |  |  |  |
| Stirred cell vs. Centrifugal ultrafilters | -20.86 | -36.60 to -5.115 | ** | 0.006 |
| Stirred cell vs. Crossflow cassettes | 0.33 | -15.41 to 16.07 | ns | 0.999 |
| Centrifugal ultrafilters vs. Crossflow cassettes | 21.19 | 5.445 to 36.93 | ** | 0.005 |
|  |  |  |  |  |
| HY 100 kDa |  |  |  |  |
| Stirred cell vs. Centrifugal ultrafilters | 5.01 | -10.73 to 20.75 | ns | 0.729 |
| Stirred cell vs. Crossflow cassettes | -16.02 | -31.76 to -0.275 | * | 0.045 |
| Centrifugal ultrafilters vs. Crossflow cassettes | -21.03 | -36.77 to -5.285 | ** | 0.006 |
|  |  |  |  |  |
| HY 300 kDa |  |  |  |  |
| Stirred cell vs. Centrifugal ultrafilters | -25.61 | -41.35 to -9.865 | *** | <0.001 |
| Stirred cell vs. Crossflow cassettes | -28.73 | -44.47 to -12.99 | *** | <0.001 |
| Centrifugal ultrafilters vs. Crossflow cassettes | -3.12 | -18.86 to 12.62 | ns | 0.884 |
